# Ninjurin1 in cardiovascular and vascular biology: From molecular mechanisms to therapeutic opportunities

**DOI:** 10.1002/ctm2.70646

**Published:** 2026-03-17

**Authors:** Muhammad Mamunur Rashid Mahib

**Affiliations:** ^1^ Department of Biochemistry and Molecular Biology University of Chittagong Chittagong Bangladesh

**Keywords:** angiogenesis, atherosclerosis, cardiovascular disease, ischaemia‒reperfusion injury, Ninjurin1, plasma membrane rupture

## Abstract

**Background:**

Ninjurin1 (NINJ1) is a transmembrane protein originally identified as a nerve injury‐induced adhesion molecule. Recent discoveries have revealed its essential role in plasma membrane rupture (PMR) during lytic cell death, positioning NINJ1 as a critical mediator at the intersection of vascular biology, inflammation, and programmed cell death. Its complex and context‐dependent biology makes it a compelling target for cardiovascular research.

**Methods:**

This review comprehensively synthesizes evidence from structural, molecular, cellular, and in vivo studies on NINJ1. We integrated data on NINJ1's structural biology, its cell‐type‐specific roles in endothelial cells, macrophages, smooth muscle cells, and pericytes, and its contributions to major cardiovascular diseases, including atherosclerosis, myocardial infarction, aortic aneurysm, and ischemia‐reperfusion injury. Emerging therapeutic strategies targeting NINJ1 oligomerization were also evaluated.

**Results:**

NINJ1 exhibits a fundamental biological paradox in cardiovascular pathophysiology. In its membrane‐bound form, NINJ1 transitions from an autoinhibited homodimer to an active polymeric filament upon cell death stimulation, executing PMR and releasing damage‐associated molecular patterns (DAMPs) that amplify vascular inflammation. In contrast, its soluble MMP‐9‐cleaved ectodomain (sNINJ1) suppresses macrophage activation, attenuates monocyte‐endothelial interactions, and exerts potent atheroprotective effects. NINJ1 is dynamically regulated across multiple cardiovascular pathologies and contributes to endothelial dysfunction, plaque instability, myocardial injury, and pericyte‐mediated vascular remodeling.

**Conclusions:**

NINJ1 is a pivotal and therapeutically tractable mediator of cardiovascular inflammation. Its dual roles in promoting PMR‐driven DAMP release and in limiting inflammation through sNINJ1 signaling provide complementary avenues for therapeutic intervention. Strategies targeting NINJ1 oligomerization or exploiting sNINJ1‐mimetic peptides hold promise for the treatment of inflammatory cardiovascular diseases and warrant further translational investigation.

**Key points:**

- Membrane‐bound NINJ1 oligomerizes into amphipathic filaments to execute plasma membrane rupture (PMR) during lytic cell death, releasing DAMPs that propagate vascular inflammation across multiple cardiovascular pathologies.
- The soluble MMP‐9‐cleaved ectodomain of NINJ1 (sNINJ1) exerts anti‐inflammatory and atheroprotective effects, creating a functional paradox in which a single protein can both promote and restrain cardiovascular inflammation.
- NINJ1 exerts cell‐type‐specific functions in endothelial cells, macrophages, smooth muscle cells and pericytes, contributing to atherosclerosis, myocardial infarction, aortic aneurysm and ischemia‐reperfusion injury.
- Targeting NINJ1 oligomerization or utilizing sNINJ1‐mimetic peptides represents a novel therapeutic strategy with potential for treating inflammatory cardiovascular diseases.

## Graphical abstract

NINJ1 mediates PMR in cardiovascular cell death, releasing DAMPs that amplify vascular inflammation.

Dual NINJ1 paradox: membrane‐bound form drives endothelial NF‐κB/CXCL‐8 inflammation while soluble form provides atheroprotection.

Pericyte NINJ1 regulates the critical balance between angiogenic sprouting and vessel maturation in ischaemic tissues.

Cell‐type‐specific NINJ1 functions reveal precision therapeutic opportunities: blocking oligomerisation while enhancing sNINJ1 generation.

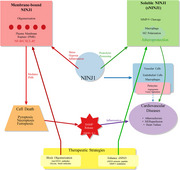

## 1 INTRODUCTION

Cardiovascular diseases (CVDs) remain the leading cause of mortality worldwide, accounting for approximately 17.9 million deaths annually.^1^ Despite significant advances in lipid‐lowering therapies and interventional procedures, substantial cardiovascular morbidity and mortality persist, driven by multiple interrelated factors including residual inflammatory risk, metabolic dysfunction, genetic susceptibility, and incompletely addressed pathophysiological mechanisms.^2^, ^3^ The recognition that inflammation plays a central pathophysiological role in atherosclerosis—from initial endothelial activation through foam cell formation to plaque rupture and thrombosis—has stimulated substantial interest in immunomodulatory therapeutic approaches for atherosclerotic CVD.^2^, ^4^ However, inflammatory mechanisms also contribute to other cardiovascular conditions, including heart failure,^5^ arrhythmias and cardiomyopathies.^6^

Ninjurin1 (NINJ1), initially identified by Araki and Milbrandt in 1996 as a novel adhesion molecule upregulated after sciatic nerve injury, has recently emerged as a critical mediator of cardiovascular pathophysiology.^7^ This 16‐kDa transmembrane protein, encoded by the NINJ1 gene on chromosome 9q22.31, was originally characterised for its role in promoting axonal growth and nerve regeneration through homophilic interactions. However, over the past decade, research has revealed NINJ1's complex and multifaceted roles in vascular biology, positioning it as a key player in endothelial function, macrophage biology, angiogenesis, and inflammatory cell death.^6^, ^7^

It is essential to distinguish NINJ1 from its paralogue Ninjurin2 (NINJ2), which shares approximately 55% sequence homology but has fundamentally different functions. While genome‐wide association studies (GWAS) have linked NINJ2 polymorphisms to ischaemic stroke risk in Asian populations (rs11833579, odds ratio 1.21, 95% confidence interval 1.12‒1.31),^8^ NINJ1 itself has distinct and more direct clinical associations with CVD. Elevated serum NINJ1 concentrations correlate with the severity of large artery atherosclerotic stroke.^9^ Increased NINJ1 protein expression is observed in human atherosclerotic plaques compared with matched healthy arterial tissue, with NINJ1 localising predominantly to plaque macrophages and foam cells.^9^ Furthermore, elevated plasma NINJ1 levels in patients with non‐valvular atrial fibrillation correlate with indices of atrial remodelling and thromboembolic risk.^10^ Critically, Sahoo et al.^11^ have definitively established that NINJ2 cannot mediate plasma membrane rupture (PMR) despite its ability to oligomerise, providing a molecular explanation for the functional divergence between these paralogues. These NINJ1‐specific clinical associations and mechanistic data should not be conflated with NINJ2 genetic studies. Subsequent investigations demonstrated that NINJ1 itself is dynamically regulated in cardiovascular pathologies and participates in critical processes, including leukocyte transmigration, vascular remodelling and inflammatory responses.^12^ More recently, groundbreaking structural studies have revealed that NINJ1 mediates PMR during lytic cell death—a discovery that has fundamentally altered our understanding of how dying cells release inflammatory mediators.^13^

This dual nature of NINJ1 presents a fascinating paradox in cardiovascular biology. On the one hand, the soluble extracellular domain of NINJ1 (sNINJ1), generated by matrix metalloproteinase‐9 (MMP‐9) cleavage, exhibits anti‐inflammatory and atheroprotective properties.^9^ On the other hand, membrane‐bound NINJ1 mediates inflammatory cell death, facilitating the release of damage‐associated molecular patterns (DAMPs) that propagate vascular inflammation.^13^, ^14^ Understanding these context‐dependent functions is crucial for developing targeted therapeutic strategies.

This review comprehensively examines the role of NINJ1 in cardiovascular and vascular biology. We discuss its structural features and mechanisms of activation, explore its cell‐type‐specific functions in endothelial cells, macrophages, smooth muscle cells and pericytes, and analyse its contributions to major cardiovascular pathologies, including atherosclerosis, myocardial ischaemia‒reperfusion injury (IRI) and diabetic vascular complications. Finally, we evaluate emerging therapeutic strategies targeting NINJ1 and outline future research directions that may translate these findings into clinical applications.

## 2 STRUCTURAL BIOLOGY AND MOLECULAR MECHANISMS OF NINJ1

### 2.1 Protein structure and topology

NINJ1 is a highly conserved transmembrane protein consisting of 152 amino acids with a molecular weight of approximately 16 kDa.^15^ Recent cryo‐electron microscopy (cryo‐EM) structural analyses have significantly revised our understanding of NINJ1 membrane topology. This necessitates a fundamental reevaluation of earlier models, which were based on biochemical and immunological approaches that suggested an extracellular N‐terminus, inferred from antibody accessibility studies and glycosylation site predictions. In its autoinhibited dimeric form—the predominant state of NINJ1 under non‐stressed conditions—NINJ1 adopts a configuration where the N‐terminus is intracellular, with both transmembrane helices (transmembrane helix 1 [TM1] and transmembrane helix 2 [TM2]) spanning the plasma membrane in an antiparallel orientation.^16^ This definitive structural determination, achieved through state‐of‐the‐art cryo‐EM that provides direct visualisation of protein architecture in a near‐native lipid environment, supersedes earlier topology models that could not achieve equivalent structural resolution. The autoinhibited homodimer is stabilised by extensive face‐to‐face interactions between the two NINJ1 protomers, which effectively sequester the amphipathic helices α1 (residues 45–65) and α2 (residues 68–85), preventing their premature membrane insertion and consequent cytotoxic oligomerisation. The hydrophilic faces of these amphipathic helices are buried at the dimer interface through complementary electrostatic interactions and hydrogen‐bonding networks, while the hydrophobic faces remain oriented towards the surrounding lipid environment of the plasma membrane. This arrangement creates a thermodynamically stable configuration that keeps NINJ1 in an inactive state under normal cellular conditions. Additionally, TM1 (residues 100–120) adopts an unkinked, straight conformation in the autoinhibited state, which is structurally incompatible with the lateral protein‒protein interactions required for filament assembly. The TM1 helix contains a conserved glycine residue (Gly109) that enables the kinked conformation required for oligomerisation; in the autoinhibited dimer, interactions across the dimer interface constrain TM1 in an extended, non‐kinked configuration. This multifaceted autoinhibition mechanism—involving both sequestration of amphipathic helices and conformational restriction of TM1—maintains NINJ1 in a catalytically inactive state until appropriate cell death signals trigger the dramatic conformational reorganisation required for PMR execution. Upon activation by cell‐death stimuli—including pyroptotic signals downstream of inflammasome activation, necroptotic signals mediated by the RIPK1/RIPK3/MLKL cascade, ferroptotic lipid peroxidation and mechanical membrane strain—NINJ1 undergoes a dramatic, essentially irreversible conformational transition. The face‐to‐face dimer interface is disrupted, exposing the previously sequestered amphipathic α1 and α2 helices. The N‐terminal region, including the previously characterised N‐terminal adhesion motif (N‐NAM, residues Pro26‐Asn37), reorients as the α1 and α2 helices spontaneously partition into the lipid bilayer, driven by the thermodynamic favourability of burying hydrophobic residues within the membrane interior. In the fully activated polymeric filament state, these helices become embedded within the membrane, with their hydrophobic faces interacting with membrane lipids and their hydrophilic faces creating membrane‐destabilising interfaces.^16^, ^17^ This remarkable structural plasticity—wherein a single protein can transition from a compact, autoinhibited homodimer to an extended, membrane‐embedded polymeric filament—reconciles the apparent paradox between NINJ1's historical characterisation as an adhesion molecule mediating homophilic cell‒cell interactions and its recently discovered function as the terminal executor of PMR during inflammatory cell death. The structural data from Pourmal et al. are methodologically rigorous and internally consistent with functional autoinhibition data; we find no compelling reasons to question their validity and explicitly endorse this structural model as the current consensus view of NINJ1 topology in the autoinhibited state.^16^

**FIGURE 1:**
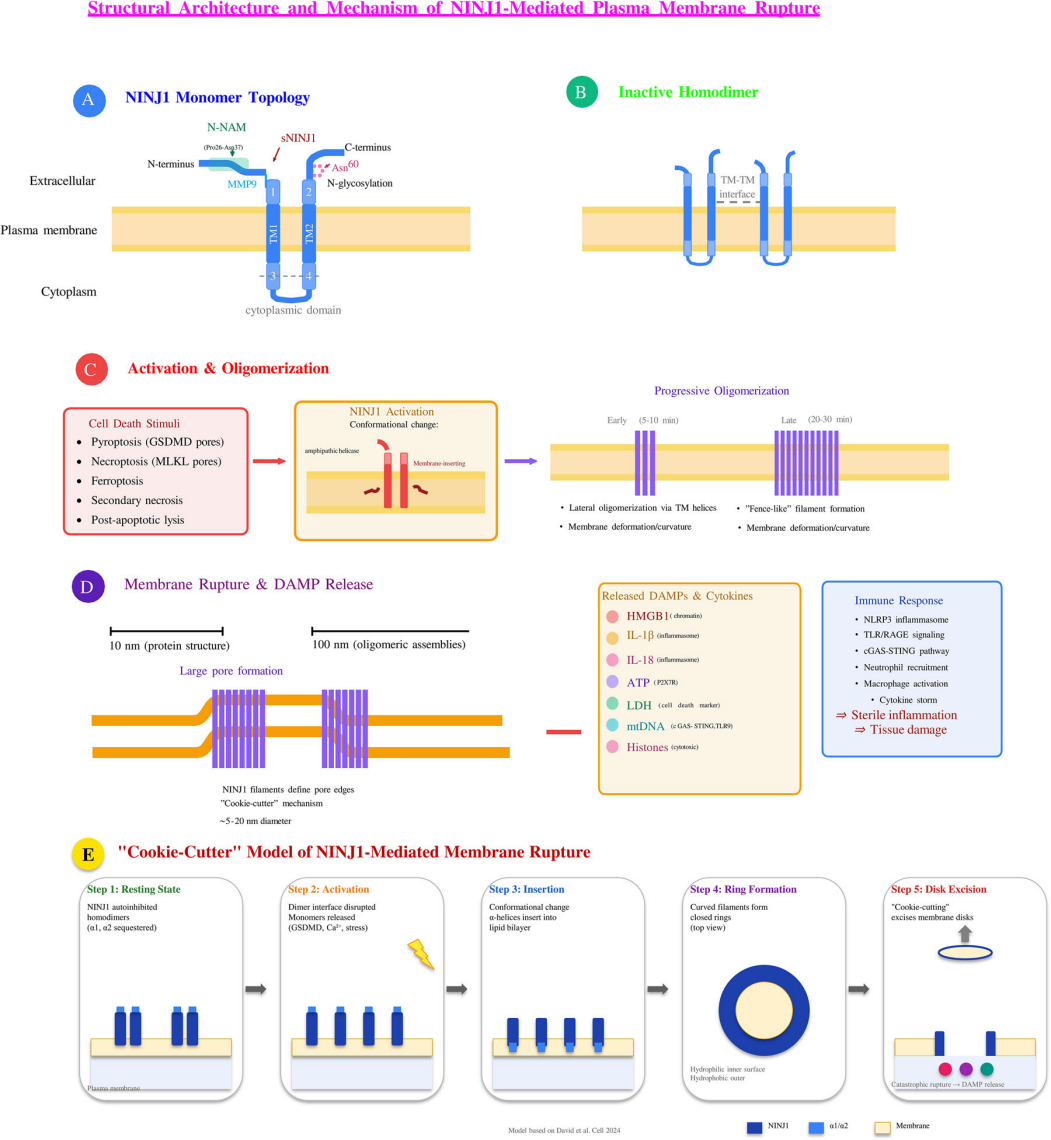
Structural architecture and mechanism of NINJ1‐mediated plasma membrane rupture. (a) Topology of NINJ1 monomer. NINJ1 (Ninjurin 1) is a 152‐amino acid transmembrane protein with an unusual hairpin topology featuring both N‐ and C‐termini on the extracellular side. The protein contains an N‐terminal N‐NAM domain (Pro26‐Asn37), two extracellular α‐helices (α1, α2), two transmembrane helices (TM1, TM2), and two cytoplasmic α‐helices (α3, α4). N‐glycosylation occurs at Asn60. Matrix metalloproteinase 9 (MMP9) cleaves the extracellular domain to generate soluble NINJ1 (sNINJ1). (b) Inactive homodimer state. In resting cells, NINJ1 exists as an autoinhibited homodimer stabilized by transmembrane helix‐helix interactions, preventing spontaneous oligomerization and membrane perturbation. (c) Cell death‐ induced activation and progressive oligomerization. Multiple cell death pathways (pyroptosis, necroptosis, ferroptosis, secondary necrosis, post‐apoptotic lysis) trigger NINJ1 conformational changes, exposing hydrophobic surfaces and promoting amphipathic helix insertion into the membrane. Activated NINJ1 undergoes lateral oligomerization via interactions within the transmembrane domain, forming progressively larger assemblies. Early oligomers (5‐10 min) grow into extensive "fence‐like" filament arrays (20‐30 min) that induce membrane deformation and curvature. (d) Plasma membrane rupture and DAMP release. NINJ1 oligomeric filaments define the edges of large membrane pores (5‐20 nm diameter) through a "cookie‐cutter" mechanism, causing plasma membrane rupture. This releases diverse damage‐associated molecular patterns (DAMPs), including HMGB1 (chromatin‐associated), IL‐1β and IL‐18 (inflammasome products), ATP (purinergic signal), LDH (cell death marker), mitochondrial DNA (mtDNA), and histones. Released DAMPs trigger robust inflammatory responses through pattern recognition receptors (NLRP3 inflammasome, TLR/RAGE, cGAS‐STING pathway), leading to neutrophil. (e) Schematic of the "cookie‐cutter" model of NINJ1‐mediated membrane rupture. In the resting state (Step 1), NINJ1 exists as autoinhibited homodimers with α1 and α2 helices sequestered. Upon cell death stimuli such as GSDMD pore formation, Ca^2^^+^ influx, or cellular stress (Step 2), the dimer interface is disrupted and monomers are released. Activated monomers undergo conformational changes that drive insertion of amphipathic α‐helices into the lipid bilayer (Step 3), with hydrophobic surfaces facing the membrane interior and hydrophilic surfaces oriented inward. Membrane‐inserted NINJ1 monomers then assemble into curved filaments that close into ring‐shaped oligomeric structures (Step 4). Finally, the closed NINJ1 rings excise membrane disks through a "cookie‐cutting" mechanism (Step 5), resulting in catastrophic plasma membrane rupture and subsequent DAMP release. Model based on David et al., *Cell* (2024)

The extracellular region of NINJ1 contains two amphipathic α‐helices (α1 and α2) (Figure 1a), that are critical for its function.^12^ These helices possess distinct hydrophobic and hydrophilic faces, a structural feature that proves essential for NINJ1's membrane‐disrupting activity.^12^, ^18^ Additionally, the extracellular N‐NAM, spanning residues Pro26 to Asn37, mediates homophilic cell‒cell interactions—a function originally associated with NINJ1's role in neural regeneration but also relevant in vascular biology.^17^, ^19^

In its inactive state, NINJ1 exists predominantly as a homodimer (Figure 1b), on the plasma membrane.^16^ This dimeric configuration involves face‐to‐face interactions that sequester the hydrophilic faces of the α1 and α2 helices, effectively maintaining autoinhibition of its membrane‐disrupting activity.^18^ In the inactive state, TM1 adopts an unkinked conformation that prevents premature oligomerisation.^16^, ^20^

### 2.2 Mechanism of oligomerisation and plasma membrane rupture

The discovery that NINJ1 mediates PMR represents one of the most significant recent advances in cell‐death biology.^13^ Upon activation by various cell‐death stimuli—including pyroptosis, necroptosis, apoptosis and ferroptosis—NINJ1 undergoes dramatic conformational changes and oligomerisation^14^, ^20^ (Figure 1c).

The activation process involves several sequential steps. First, death signals disrupt the inhibitory homodimer, allowing NINJ1 to transition to an active, four‐helix‐kinked conformation.^17^ In this activated state, the extracellular α1 and α2 helices insert into the plasma membrane lipid bilayer. This membrane insertion is driven by the amphipathic nature of these helices, with their hydrophobic faces interacting with membrane lipids while their hydrophilic faces remain exposed.^12^, ^13^

Following activation, NINJ1 monomers polymerise into higher‐order oligomeric structures (Figure 1d). Time‐course crosslinking experiments have demonstrated progressive NINJ1 oligomerisation, with dimers and trimers forming within 10 min of inflammasome activation, followed by extensive polymerisation into large oligomeric complexes.^12^ Super‐resolution microscopy has revealed that these oligomers assemble into structurally diverse forms, including large, branched filamentous assemblies.^12^

Cryo‐EM structural analysis has shown that NINJ1 filaments form a tightly packed fence‐like array of transmembrane α‐helices.^12^, ^21^ These filaments exhibit distinct structural asymmetry, with a hydrophilic side and a hydrophobic side. The directionality and stability of filaments are defined by two amphipathic α‐helices (α3 and α4 in the intracellular region) that interlink adjacent filament subunits.^22^ Molecular dynamics simulations have demonstrated that these filaments can stably cap membrane edges, providing a mechanistic explanation for membrane disruption.^13^

Three distinct mechanistic models have been proposed to explain how oligomerised NINJ1 executes PMR, each supported by complementary structural and functional evidence (Table 1). A systematic comparison of these models—including their core mechanisms, structural bases, key evidence, similarities, differences and therapeutic implications—is essential for understanding NINJ1 biology and for rational therapeutic development. The first model, termed the ‘membrane edge‐capping’ mechanism, was proposed by Degen et al. based on cryo‐EM structural analysis of NINJ1 filaments.^17^ In this model, NINJ1 assembles into amphipathic polymeric filaments that preferentially localise to membrane edges generated by upstream membrane‐damaging events, such as gasdermin D (GSDMD) pore formation or mixed lineage kinase domain‐like protein (MLKL) insertion. The structural basis derives from cryo‐EM reconstructions revealing NINJ1 filaments with a distinctive fence‐like architecture, featuring clear segregation of hydrophobic (membrane‐facing) and hydrophilic (aqueous‐facing) surfaces, and filament geometry compatible with binding to membrane edges, where both lipid headgroups and acyl chains are exposed. Rather than actively creating membrane lesions, NINJ1 in this model functions through passive stabilisation: by coating the edges of membrane wounds, NINJ1 oligomers prevent the normal membrane repair processes mediated by Endosomal Sorting Complexes Required for Transport (ESCRT) machinery and calcium‐dependent repair pathways that would otherwise reseal membrane damage. Progressive accumulation of unrepaired lesions ultimately leads to catastrophic membrane failure and complete cellular lysis. The second model, termed the ‘cookie‐cutter’ or ‘Ninja Cutter’ mechanism, was advanced by David et al.^12^ and proposes an active membrane fragmentation process. In this model, NINJ1 filaments actively form closed circular rings that physically excise circular membrane discs, analogous to a molecular cookie cutter punching out dough. The structural basis includes cryo‐EM evidence of ring‐shaped NINJ1 oligomers with curvature consistent with ring closure, and specific protein‒protein interactions that favour ring closure over indefinite linear extension. Once a NINJ1 ring encloses a membrane domain, the hydrophilic inner surface of the ring creates an energetically unfavourable environment for the enclosed lipids, thereby driving disc excision and release. Key supporting evidence includes the biochemical detection of released membranous vesicles (the excised ‘discs’) during NINJ1‐mediated lysis and tomographic reconstructions showing the apparent liberation of membrane discs. Each excision event directly reduces membrane surface area, and progressive ring formation leads to membrane fragmentation and eventual catastrophic failure. The third model, contributed by Sahoo et al., addresses a distinct but equally fundamental question: the structural basis for the functional divergence between NINJ1 and its homologue, NINJ2.^11^ Despite sharing approximately 55% sequence identity and conserved two‐pass transmembrane architecture, only NINJ1 can execute PMR‒NINJ2 is completely unable to rupture membranes despite structural similarity. Through systematic mutagenesis, chimeric protein analysis, and comparative structural modelling, Sahoo et al. identified specific residues within the amphipathic α1 and α2 helices—including key hydrophobic amino acids at positions critical for membrane interaction—that confer unique membrane‐destabilising properties to NINJ1 that are absent in NINJ2. These NINJ1‐specific residues affect the depth and angle of membrane insertion and create a more potent hydrophobic surface for membrane destabilisation. This model explains why NINJ2 cannot substitute for NINJ1 in PMR and why genetic associations with NINJ2 should not be extrapolated to NINJ1 function. Common features across all three models include: (1) the absolute requirement for NINJ1 oligomerisation/polymerisation—monomeric or dimeric NINJ1 is inactive and cannot execute PMR; (2) the necessity of amphipathic helix membrane insertion as a critical mechanistic step; (3) positioning of PMR as a terminal, downstream event occurring after upstream death signalling and initial membrane permeabilisation; (4) formation of higher‐order assemblies (filaments, rings or both) within the membrane; and (5) essential irreversibility once sufficient oligomerisation has occurred, explaining the ‘point of no return’ in lytic cell death. The fundamental distinction between the Degen and David models lies in passive versus active membrane disruption: the edge‐capping model proposes that NINJ1 prevents membrane repair (passive), while the cookie‐cutter model proposes that NINJ1 physically excises membrane material (active). These models are not necessarily mutually exclusive—NINJ1 may employ different mechanisms depending on cellular context, upstream death signals or stage of the lysis process. The Sahoo model is complementary to both, addressing molecular specificity rather than mechanism per se. Therapeutic implications of these mechanistic insights are substantial. Oligomerisation inhibitors—including antibodies that sterically block filament assembly (such as the D1 antibody binding to the C‐terminus)^23^ or small molecules that stabilise the autoinhibited dimer—should be effective across all mechanistic models, as all require oligomerisation as an essential step. Strategies that stabilise the autoinhibited dimer by reinforcing the face‐to‐face interface revealed by Pourmal et al. would prevent all downstream mechanisms by maintaining NINJ1 in its inactive state. The Sahoo model has important implications: it demonstrates that NINJ2 cannot compensate for NINJ1 inhibition, eliminating concerns about functional redundancy between paralogues and supporting NINJ1 as a druggable target.^11^, ^12^, ^17^

**TABLE 1:** Comparison of proposed mechanistic models for Ninjurin1 (NINJ1)‐mediated plasma membrane rupture.

Feature	Degen et al.^17^	David et al.^12^	Sahoo et al.^11^/Pourmal et al.^16^
Model name	Pore‐forming model	Cookie‐cutter model (‘Ninja Cutter’)	Refined cookie‐cutter with N‐NAM topology revision
Primary structural method	Cryo‐EM of NINJ1 filaments from liposomes	Cryo‐EM of ring segments from pyroptotic cells	Cryo‐EM of full‐length NINJ1; X‐ray crystallography
N‐terminus (N‐NAM) topology	Extracellular (predicted)	Extracellular (predicted)	Intracellular (experimentally validated)
Inactive state structure	Not determined	Not determined	Face‐to‐face autoinhibited homodimer via amphipathic helices
Activation trigger	Downstream of GSDMD pores; mechanism unclear	Membrane permeabilisation, lipid redistribution, ionic influx	Cell swelling/mechanical strain; dimer interface disruption
Oligomerisation mechanism	Linear filament assembly via TM helix interactions	Curved filaments forming closed rings; TM helices kinked	Dimer dissociation → monomer membrane insertion → filament/ring assembly
PMR mechanism	Pore formation similar to other membrane proteins	Ring closure excises membrane disks; lesions of variable size	Cookie‐cutter excision; explains heterogeneous lesion sizes
Critical residues identified	TM helix residues for filament contacts	Gly93, Gly95, Gly124 (TM kinks); Lys cluster for membrane localisation	Lys45‒Asp53 salt bridge; Ala59 (human) versus Thr59 (mouse); Asn60 glycosylation
Supporting evidence	Filament structures resolved Liposome permeabilisation assays Mutagenesis studies	Live‐cell NINJ1‐GFP imaging Membrane disk release observed Ring structures in cryo‐EM Explains size‐unrestricted release	Protease protection assays N‐glycosylation site mapping Crystal structures of autoinhibited dimer Cell swelling activation studies
Limitations/gaps	Inactive state unknown Activation mechanism unclear N‐terminus topology incorrect	Inactive state unknown Ring closure mechanism unclear N‐terminus topology incorrect	How N‐NAM mediates adhesion if intracellular? In vivo validation needed Species differences in activation
Therapeutic implications	Target TM helix interactions to prevent filament assembly	Target ring closure or membrane disk excision	Target dimer interface to prevent activation; exploit species differences

*Note*: This table summarises the three major structural models proposed for NINJ1‐mediated PMR. The field has evolved from initial pore‐forming models (Degen et al.) through the cookie‐cutter mechanism (David et al.) to refined models that correct the N‐terminal topology (Sahoo et al./Pourmal et al.). All models agree that NINJ1 oligomerisation is essential for PMR and that this process occurs downstream of GSDMD pore formation during pyroptosis. The most significant recent revision is the demonstration that the N‐terminus, which contains the adhesion motif (N‐NAM), is intracellular rather than extracellular, raising important questions about how the historically attributed adhesion functions of NINJ1 are mediated.

Abbreviations: Cryo‐EM, cryo‐electron microscopy; GSDMD, gasdermin D; PMR, plasma membrane rupture; TM, transmembrane.

#### 2.2.1 Critical distinction: NINJ1‐mediated membrane disruption versus gasdermin pore formation

It is crucial to clearly distinguish NINJ1‐mediated membrane disruption from that of canonical pore‐forming proteins such as GSDMD, as these mechanisms are fundamentally different and should not be conflated, even though both contribute to inflammatory cell death (Table 2). GSDMD, after caspase‐1‐mediated cleavage, releases the N‐terminal domain (GSDMD‐NT) from its autoinhibitory C‐terminal domain, forming well‐defined annular pores consisting of 24–33 subunits arranged in a symmetric β‐barrel structure.^24^, ^25^ These GSDMD pores have stable inner diameters of approximately 18–21 nm, shaped by the fixed stoichiometry and geometry of the β‐barrel structure. Importantly, GSDMD pores are selectively permeable based on molecule size and charge, allowing the passage of specific molecules—including mature IL‐1β (∼17 kDa), IL‐18 (∼18 kDa), potassium ions, and adenosine triphosphate (ATP)—while blocking larger intracellular proteins such as lactate dehydrogenase (LDH, ∼140 kDa tetramer) and high mobility group box 1 (HMGB1, ∼25 kDa). Significantly, GSDMD pore formation preserves overall plasma membrane integrity: cells can survive for extended periods (minutes to hours) with these pores, actively releasing cytokines through them while maintaining viability, metabolic function and responsiveness to external signals. Evidence indicates that the ESCRT machinery can repair GSDMD pores, thereby supporting cell survival. In sharp contrast, NINJ1 forms structurally diverse, open‐ended filamentous assemblies that vary considerably in length, can be linear, curved or branched, and lack a defined pore size or fixed stoichiometry.^12^, ^17^ No two NINJ1 oligomers are structurally identical—the assemblies are fundamentally heterogeneous. Rather than forming stable conduits for selective molecular passage, NINJ1 oligomerisation culminates in catastrophic membrane disintegration and the complete loss of cellular compartmentalisation. NINJ1‐mediated PMR is entirely non‐selective: membrane rupture releases all intracellular contents, regardless of molecular weight, including small ions, cytokines, large proteins (HMGB1 and LDH) and even organellar contents. There is no ‘survival with NINJ1 activation’—once sufficient NINJ1 oligomerisation has occurred and PMR is initiated, the process is terminal and irreversible. This mechanistic distinction explains the observed temporal sequence during pyroptosis, the best‐characterised context for NINJ1 function: GSDMD pores form early after inflammasome activation (within minutes of caspase‐1 activation), enabling selective ion flux (potassium efflux driving further inflammasome activation, calcium influx), cell swelling due to osmotic water entry, and selective cytokine release (IL‐1β and IL‐18). NINJ1‐mediated PMR occurs subsequently as the terminal execution event (typically delayed by minutes to hours, depending on cellular conditions, NINJ1 expression levels and upstream signal intensity), releasing the full spectrum of intracellular DAMPs, including HMGB1 and LDH, which drive sterile inflammatory responses in surrounding tissue.^13^ This temporal separation has therapeutic implications: targeting GSDMD inhibits early cytokine release while potentially preserving cell viability, whereas targeting NINJ1 specifically prevents terminal membrane rupture and large DAMP release while permitting GSDMD‐mediated selective cytokine secretion. Combination strategies targeting both proteins may provide comprehensive control of inflammatory cell death.

**TABLE 2:** Mechanistic distinctions between gasdermin D (GSDMD) and Ninjurin1 (NINJ1) in pyroptotic membrane disruption.

Parameter	GSDMD	NINJ1
Stoichiometry	24‒33 subunits forming defined oligomeric pores	Variable stoichiometry; no fixed subunit number in filament assemblies
Structure	Symmetric β‐barrel architecture with organised pore geometry	Heterogeneous α‐helical filaments with irregular membrane associations
Diameter	Consistent 18–21 nm pore diameter	Undefined; causes progressive membrane fragmentation rather than defined channels
Selectivity	Size‐ and charge‐selective permeabilisation (allows specific molecules based on properties)	Non‐selective membrane rupture; complete cellular content release
Mechanism	Pore formation with maintained membrane integrity around organised structures	Membrane fragmentation and complete rupture; loss of membrane integrity
Reversibility	Potentially repairable by ESCRT‐III machinery	Irreversible; terminal membrane destruction
Temporal relationship	Early event in pyroptosis (minutes after inflammasome activation)	Late/terminal event (follows GSDMD pore formation)

*Note*: GSDMD quickly forms structured, size‐selective pores that enable controlled cytokine release while preserving membrane integrity. Conversely, NINJ1 induces late‐stage, irreversible membrane fragmentation characterised by irregular structures, non‐selective rupture and loss of total cellular content. These proteins operate in distinct temporal and mechanistic phases of pyroptotic cell death: GSDMD triggers inflammation through regulated pore formation, whereas NINJ1 concludes the process with catastrophic membrane disruption leading to cell lysis.

#### 2.2.2 Molecular triggers of NINJ1 polymerisation: from biochemical signals to mechanical strain

The mechanisms that trigger NINJ1's transition from its autoinhibited dimeric state to the active oligomeric form have recently been elucidated through complementary biochemical and biophysical studies, representing a major advance in understanding how upstream cell‐death signals are transduced into NINJ1 activation. These insights are particularly relevant for cardiovascular biology, where cells are continuously exposed to both biochemical inflammatory signals and mechanical forces. Dondelinger et al. systematically investigated the biochemical triggers that converge on NINJ1 activation during inflammatory cell death.^26^ Their studies demonstrated that upstream cell‐death signalling cascades induce a conformational change in NINJ1 through coordinated alterations in the plasma membrane environment and the intracellular ionic milieu. Key biochemical triggers identified include: (1) loss of plasma membrane phospholipid asymmetry, characterised by phosphatidylserine (PS) externalisation from the inner to outer membrane leaflet. In healthy cells, PS is actively maintained on the cytoplasmic leaflet by flippases (ATP11A and ATP11C). During cell death, caspase‐mediated inactivation of flippases combined with activation of scramblases (TMEM16F/ANO6 and Xkr8) causes PS externalisation, creating conditions permissive for NINJ1 activation. (2) Elevation of intracellular calcium concentrations, a common feature of multiple cell‐death pathways, including pyroptosis (where GSDMD pores allow calcium entry), necroptosis (where MLKL channels permit calcium flux) and general membrane damage. Elevated cytosolic calcium may directly influence NINJ1 or activate calcium‐dependent enzymes (calpains and phospholipases) that modify the membrane environment. (3) Accumulation of reactive oxygen species (ROS), which may modify NINJ1 through oxidation of cysteine residues affecting protein conformation or through lipid peroxidation, altering membrane properties. (4) Intracellular acidification due to lactate accumulation, proton leak from damaged organelles, and loss of ion homeostasis during cell death. Particularly relevant to cardiovascular biology, Zhu et al. reported that mechanical membrane strain can directly trigger NINJ1 activation, independent of canonical cell‐death biochemical signalling pathways.^27^ Their elegant biophysical studies demonstrated that plasma membrane tension above a critical threshold (∼3‒5 mN/m, though cell‐type dependent) induces NINJ1 oligomerisation even in the complete absence of inflammasome activation, GSDMD pore formation, necroptotic signalling or classical apoptotic cascades. The proposed mechanism involves strain‐induced conformational changes that destabilise the autoinhibited dimer by disrupting the face‐to‐face interactions that normally sequester the amphipathic helices. The autoinhibited dimer may be stabilised by specific lipid interactions that are perturbed when membrane tension increases, lowering the activation energy barrier for conformational change. This mechanosensitive property of NINJ1 has profound implications for cardiovascular pathophysiology, as cells in the cardiovascular system experience continuous mechanical forces that vary dramatically under physiological and pathological conditions: in systemic hypertension, chronically elevated blood pressure increases transmural pressure across vessel walls, subjecting endothelial cells and vascular smooth muscle cells (VSMCs) to increased membrane strain. This may lower the threshold for NINJ1 activation in response to additional inflammatory stressors, potentially explaining the well‐documented association between hypertension and vascular inflammation that extends beyond purely haemodynamic effects. The observation that blood pressure reduction decreases cardiovascular events to a greater degree than predicted by haemodynamic modelling alone may partially reflect reduced mechanical priming of NINJ1‐mediated inflammatory cell death. At atherosclerotic plaque shoulders, complex flow patterns create regions of disturbed haemodynamics characterised by low and oscillatory shear stress. These regions experience increased membrane mechanical stress due to flow separation and reattachment, pressure differentials across stenotic segments, and turbulent eddies impinging on the endothelial surface. Remarkably, these mechanically stressed regions precisely correspond to the locations where plaque inflammation is highest and rupture risk is greatest. The correlation between zones of pathological mechanical stress and inflammatory hot spots within plaques may reflect mechanically triggered NINJ1 activation in endothelial cells and plaque macrophages, thereby contributing to the focal distribution of plaque destabilisation. In an aortic aneurysm, where the vessel diameter is pathologically increased, wall tension rises dramatically according to Laplace's Law (wall tension = transmural pressure × vessel radius). VSMCs and infiltrating macrophages in aneurysmal segments, therefore, experience substantially elevated mechanical stress. This creates conditions for mechanically triggered NINJ1 activation, potentially establishing a devastating feed‐forward loop: initial wall weakening leads to vessel dilation, which increases radius and therefore wall tension per Laplace's Law; elevated tension triggers NINJ1 activation in medial cells; NINJ1‐mediated PMR releases DAMPs (HMGB1 and IL‐1α) that activate matrix metalloproteinases (MMP‐2, MMP‐9 and MMP‐12); MMP activity degrades elastic fibres and collagen causing further wall weakening; this permits additional dilation, completing the destructive cycle. This mechanistic model explains both the progressive nature of aneurysm expansion and the clinical observation that inflammation correlates with aneurysm growth rate. During cardiac IRI, cardiomyocytes swell due to ion pump failure (inhibition of Na^+^/K^+^‐ATPase during ischaemia), thereby increasing membrane strain. Upon reperfusion, rapid re‐energisation causes additional osmotic stress, and calcium overload induces contracture against the swollen cell state, creating mechanical strain that may trigger NINJ1 independently of classical death pathways. This could explain why some cardiomyocyte death during ischaemia‒reperfusion cannot be fully prevented by inhibiting individual death pathways. In heart failure with volume overload, cardiac chambers dilate and cardiomyocytes experience chronic stretch with elevated membrane tension during diastolic filling, potentially contributing to ongoing cardiomyocyte loss that drives disease progression. These findings position NINJ1 as a molecular mechanosensor that directly links pathological mechanical forces to inflammatory cell‐death responses in the cardiovascular system—a conceptual advance with significant implications for understanding how haemodynamic factors contribute to the pathogenesis and progression of CVD.^26^, ^27^

#### 2.2.3 Molecular mechanism of the dimer‐to‐polymer transition

The transition from the autoinhibited NINJ1 homodimer to the membrane‐disrupting polymeric state represents one of the most dramatic conformational changes known in membrane protein biology. Understanding the structural and energetic basis of this transition is not only scientifically important but also therapeutically critical, as this conformational switch represents a key intervention point where pharmacological stabilisation of the inactive state could prevent inflammatory cell death. In its inactive state, NINJ1 exists as an autoinhibited homodimer wherein extensive face‐to‐face interactions between the two protomers sequester the amphipathic α1 (residues 45–65) and α2 (residues 68–85) helices.^16^ The dimer interface buries the hydrophilic faces of these helices through complementary electrostatic interactions (including salt bridges between charged residues) and an extensive hydrogen‐bonding network that spans approximately 1500 Å^2^ of buried surface area. Additionally, TM1 (residues 100–120) adopts an unkinked, straight conformation in the inactive state that is structurally incompatible with the lateral protein‒protein interactions required for filament assembly. This unkinked TM1 configuration is enforced by cross‐dimer interactions that constrain the helix geometry. The combination of helix sequestration and TM1 conformational restriction creates a thermodynamically stable, kinetically trapped autoinhibited state with a substantial activation‐energy barrier that prevents spontaneous activation under normal cellular conditions. The transition from this autoinhibited state to the polymeric form proceeds through a defined multi‐step mechanistic sequence:

Step 1—trigger sensing (seconds to minutes): cell‐death signals—whether biochemical (calcium influx, PS externalisation, ROS accumulation, intracellular acidification) or biophysical (membrane strain exceeding adaptive thresholds)—alter the local membrane environment surrounding NINJ1 and/or directly modify the protein. These perturbations are sensed through mechanisms that may include lipid‐binding residues detecting altered membrane composition, strain‐sensitive regions in the transmembrane helices responding to membrane tension, or direct sensitivity of the dimer interface to membrane biophysical properties. The exact molecular ‘sensor’ within NINJ1 that detects these changes remains to be definitively identified and represents an important area for future investigation.

Step 2—dimer destabilisation (seconds): trigger signals alter the local environment and/or directly modify NINJ1, thereby progressively weakening the face‐to‐face dimer interface. Key stabilising interactions—including the hydrogen bonds between hydrophilic residues on the α1 and α2 helix faces, hydrophobic interactions between TM helices, and electrostatic interactions at the dimer interface—are sequentially disrupted. The activation energy barrier separating the autoinhibited state from the active state is effectively lowered by these perturbations.

Step 3—dimer dissociation (milliseconds to seconds): complete separation of the two protomers occurs when the cumulative destabilising forces exceed the remaining dimer‒interface interactions. This dissociation event has immediate and profound consequences: each liberated monomer now has EXPOSED amphipathic helices, with the hydrophobic faces of α1 and α2 presented to the aqueous cytoplasm. This is an energetically highly unfavourable state—the hydrophobic exposure penalty is estimated at approximately 25 kcal/mol per helix based on standard thermodynamic parameters—which drives the system rapidly towards the next step to relieve this energetic burden.

Step 4—membrane insertion (milliseconds): the exposed amphipathic helices spontaneously and rapidly partition into the lipid bilayer, driven by the substantial thermodynamic favourability of burying hydrophobic residues within the membrane hydrophobic core (the hydrophobic effect). The hydrophobic faces of α1 and α2 insert into the membrane interior while their hydrophilic faces remain accessible to the aqueous environment. This insertion event is essentially irreversible under physiological conditions—once the helices are membrane embedded, extracting them back to the aqueous phase would require overcoming the same ∼25 kcal/mol energetic penalty. Concurrently with membrane insertion, TM1 undergoes a critical conformational change from its unkinked (straight) to a kinked (bent) configuration, enabled by flexibility at the conserved Gly109 residue. This kinked conformation creates new lateral interaction surfaces that are essential for subsequent oligomerisation.

Step 5—lateral oligomerisation (seconds to minutes): membrane‐inserted NINJ1 monomers encounter other activated monomers through lateral diffusion within the two‐dimensional membrane environment and form oligomeric assemblies. The kinked TM1 configuration enables lateral protein‒protein interactions that are not possible in the autoinhibited state; specific residues in the kinked TM1 region form the primary oligomerisation interface. Additionally, intracellular amphipathic helices α3 and α4, which were also constrained in the dimeric state, now link adjacent filament subunits, further stabilising the growing polymer. This oligomerisation process displays positive cooperativity: initial nucleation (bringing together the first few monomers) may be rate limiting, but once a nucleus forms, additional monomers add more rapidly due to the increasing number of potential binding sites on the growing filament. This cooperative behaviour explains the threshold‐like, switch‐like activation of NINJ1.

Step 6—the ‘point of no return’ (critical transition): a defining feature of NINJ1 activation is its essential irreversibility once sufficient oligomerisation has occurred. Below a critical activation threshold, isolated activated monomers may return to an inactive state (possibly re‐forming dimers); above this threshold, the population shifts irreversibly towards oligomeric forms because each oligomerisation step is thermodynamically favourable and disassembly would require the energetically prohibitive re‐exposure of hydrophobic surfaces to the aqueous environment. This threshold behaviour, which may be cell type and context dependent, defines a ‘point of no return’ in lytic cell death. Understanding and potentially raising this threshold represents a therapeutic opportunity: interventions must act before this critical point to effectively prevent PMR.

Step 7—membrane rupture (seconds to minutes after sufficient oligomerisation): depending on the operative mechanism (edge‐capping vs. cookie‐cutter), mature NINJ1 assemblies cause membrane failure through either accumulation of unrepaired damage exceeding a critical threshold (edge‐capping model) or excision of sufficient membrane area through ring formation (cookie‐cutter model). Once membrane rupture begins, it propagates rapidly and is completely irreversible, releasing all cellular contents. The complete transition from initial trigger sensing to membrane rupture typically occurs over minutes to tens of minutes, though this timeline varies substantially depending on cell type, NINJ1 expression level, trigger intensity and cellular repair capacity.^16^, ^17^

### 2.3 Regulation of NINJ1 activity

**FIGURE 2:**
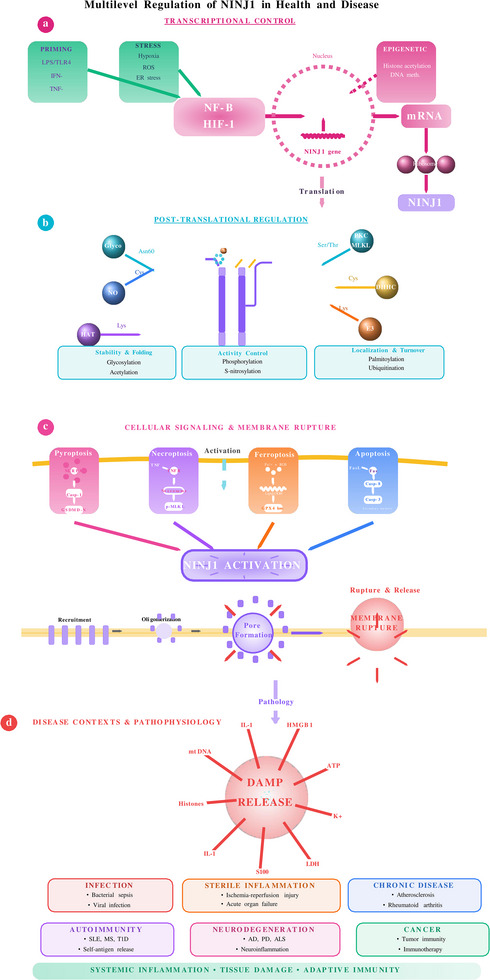
Multiple regulation of NINJ1‐mediated plasma membrane rupture in health and disease. (a)Transcriptional control of NINJ1 expression. Priming signals (LPS, IFN‐, TNF‐) and cellular stress conditions (hypoxia, ROS, ER stress) activate transcription factors, including NF‐B and HIF‐1, which drive NINJ1 gene transcription in the nucleus. Epigenetic modifications, including histone acetylation and DNA methylation, modulate transcriptional accessibility. mRNA is exported to the cytoplasm for ribosomal translation, producing nascent NINJ1 protein. (b) Post‐translational modifications regulate NINJ1 protein stability, activity, and localization. N‐glycosylation at Asn60 promotes proper protein folding and stability in the endoplasmic reticulum. Phosphorylation by PKC and MLKL at Ser/Thr residues modulates oligomerization capacity and pore‐forming activity. Palmitoylation by DHHC acyltransferases at Cys residues targets NINJ1 to lipid rafts and specific membrane domains. Ubiquitination by E3 ligases at Lys residues marks NINJ1 for proteasomal degradation, controlling protein turnover. S‐nitrosylation and acetylation provide additional regulatory layers affecting protein conformation and function. (c) Cellular signaling pathways converge on NINJ1 to execute plasma membrane rupture. Four distinct cell death modalities—pyroptosis (NLRP3 inflammasome → Caspase‐1 → GSDMD), necroptosis (TNF receptor → necrosome → phospho‐MLKL), ferroptosis (iron/ROS → lipid peroxidation → GPX4 loss), and apoptosis (death receptor → Caspase‐8 → Caspase‐3)—activate NINJ1 through distinct upstream signals. Activated NINJ1 undergoes progressive oligomerization, forming higher‐order structures that insert into the plasma membrane. These oligomers assemble into membrane‐spanning pores that cause catastrophic plasma membrane rupture, releasing intracellular contents, including damage‐associated molecular patterns (DAMPs). (d) Disease contexts and pathophysiological consequences of NINJ1‐mediated membrane rupture. DAMP release (ATP, HMGB1, IL‐1, mitochondrial DNA, histones, S100 proteins, LDH, K^+^) triggers diverse pathological conditions. In infectious diseases, excessive NINJ1 activation during bacterial sepsis or viral infections amplifies inflammatory responses. Sterile inflammation driven by NINJ1 contributes to ischemia‐reperfusion injury and acute organ failure. Chronic diseases, including atherosclerosis and rheumatoid arthritis, are exacerbated by sustained low‐level NINJ1 activity. In autoimmune disorders (systemic lupus erythematosus, multiple sclerosis, type 1 diabetes), NINJ1‐mediated release of self‐ antigens perpetuates autoimmunity. Neurodegenerative diseases (Alzheimer's disease, Parkinson's disease, amyotrophic lateral sclerosis) feature NINJ1‐dependent neuroinflammation. In cancer, NINJ1 plays dual roles affecting tumor immunity and immunotherapy responses. These pathways converge on systemic inflammation, tissue damage, and adaptive immune activation, establishing NINJ1 as a central therapeutic target across multiple disease contexts.

NINJ1 activity is regulated at multiple levels, including transcriptional control, post‐translational modifications and proteolytic processing. At the transcriptional level, NINJ1 expression is upregulated by various stress conditions (Figure 2a), including hypoxia, inflammatory cytokines such as tumour necrosis factor‐α (TNF‐α) and tissue injury.^24^, ^25^ In vascular cells, hypoxic conditions significantly enhance NINJ1 expression, suggesting its involvement in ischaemic CVDs.^26^

#### 2.3.1 Post‐translational regulation of NINJ1: glycosylation, proteolytic processing and the MMP‐9/sNINJ1 axis in cardiovascular inflammation

Post‐translational regulation of NINJ1 involves both glycosylation and proteolytic processing, although recent structural insights necessitate a critical re‐evaluation of earlier biochemical findings and highlight unresolved questions that we discuss in detail to enable readers to synthesise this complex literature.

#### 2.3.2 Asparagine‐60 glycosylation paradox

N‐linked glycosylation at Asn60 has been reported to regulate NINJ1 complex assembly and membrane localisation, based on multiple lines of evidence, including the endoglycosidase H sensitivity of NINJ1, the effects of tunicamycin (an N‐linked glycosylation inhibitor) treatment on NINJ1 molecular weight and function, and altered electrophoretic mobility upon Asn60 mutation.^28^ However, the recent structural determination by Pourmal et al., positioning the N‐terminus intracellularly in the autoinhibited dimer, raises significant questions about the compatibility of these findings with current structural models.^16^ N‐linked glycosylation is catalysed by oligosaccharyltransferase complexes operating exclusively in the endoplasmic reticulum lumen, requiring the target asparagine (within an Asn‐X‐Ser/Thr sequon) to be luminally oriented—topologically equivalent to the extracellular space for plasma membrane proteins. If Asn60 resides in an intracellular/cytoplasmic region in the mature protein, as the Pourmal et al. structure indicates, this presents a fundamental topological paradox: how can a cytoplasmically oriented residue be glycosylated by ER‐luminal machinery? Several non‐mutually exclusive hypotheses may reconcile this discrepancy: (i) glycosylation may occur during biosynthesis before NINJ1 achieves its final membrane topology. During co‐translational membrane insertion in the ER, nascent NINJ1 may transiently adopt a different topology with the N‐terminal region initially translocated into the ER lumen, allowing glycosylation, followed by subsequent conformational maturation that inverts the N‐terminus to its final intracellular position. While unprecedented, some membrane proteins do undergo post‐insertional topology changes (topogenesis). (ii) NINJ1 may exist in multiple topological states in vivo, with the cryo‐EM structure representing the predominant but not exclusive form; a subpopulation with an extracellular N‐terminus could be glycosylated and have distinct functions. (iii) The original glycosylation site assignment may require re‐evaluation—mutational studies can be confounded by indirect effects on protein folding, and definitive mass spectrometric identification of the glycopeptide is needed to confirm the site. (iv) Static cryo‐EM structures may not capture all conformational states present in living cells. We explicitly acknowledge this as an unresolved paradox requiring future investigation through direct mass spectrometric glycopeptide identification, live‐cell topology studies using membrane‐impermeant probes, and investigation of potential topogenesis mechanisms during NINJ1 biosynthesis.

#### 2.3.3 MMP‐9‐mediated sNINJ1 generation: expanded cardiovascular context

Proteolytic cleavage by MMP‐9 (also known as gelatinase B or 92‐kDa type IV collagenase) generates soluble NINJ1 (sNINJ1) through cleavage between Leu56 and Leu57.^29^ This proteolytic processing has profound implications for cardiovascular inflammation because MMP‐9 is among the most abundantly and dynamically expressed metalloproteinases in the cardiovascular system under inflammatory conditions. In atherosclerotic plaques, activated macrophages and lipid‐laden foam cells are major cellular sources of MMP‐9.^30^, ^31^ MMP‐9 expression is highest at plaque shoulders—the mechanically stressed, inflammation‐rich regions that are the predominant sites of plaque rupture.^32^ MMP‐9 activity in plaques correlates with histological features of plaque instability and clinical event risk, positioning MMP‐9 as both a pathogenic factor and potential biomarker. During acute myocardial infarction, MMP‐9 expression increases dramatically in a characteristic temporal pattern, peaking at 2–7 days post‐infarction, coinciding with maximal inflammatory cell infiltration into the infarct zone.^33^, ^34^ Cellular sources include infiltrating neutrophils (which store pre‐formed MMP‐9 in gelatinase/tertiary granules for rapid release upon activation), recruited monocytes/macrophages and activated resident cardiac fibroblasts. Elevated MMP‐9 during this inflammatory phase correlates with adverse left ventricular remodelling, chamber dilation and progression to heart failure, establishing MMP‐9 as a prognostic marker and potential therapeutic target. Neutrophil‐derived MMP‐9 represents the fastest‐responding source at sites of acute cardiovascular inflammation.^35^ Neutrophils arrive within hours of myocardial injury or plaque disruption and rapidly degranulate, releasing stored MMP‐9 without requiring de novo protein synthesis. This rapid MMP‐9 release may initiate sNINJ1 generation in the earliest phases of cardiovascular inflammatory responses. In aortic aneurysm, MMP‐9 is critically implicated in the pathological matrix degradation that drives wall weakening and progressive dilation.^36^, ^37^ MMP‐9 knockout mice are substantially protected from experimental aneurysm formation, demonstrating a causal role. The abundant MMP‐9 in aneurysmal tissue creates conditions for extensive sNINJ1 generation. The resulting sNINJ1 fragment exhibits anti‐inflammatory properties that stand in striking contrast to the pro‐inflammatory effects of membrane‐bound NINJ1‐mediated PMR: sNINJ1 suppresses macrophage inflammatory activation through inhibition of NF‐κB and MAPK signalling pathways, promotes macrophage polarisation towards the M2‐like anti‐inflammatory/reparative phenotype, and reduces expression of pro‐inflammatory cytokines (TNF‐α, IL‐6 and IL‐1β) and chemokines (MCP‐1/CCL2).^9^ This functional dichotomy suggests that MMP‐9‐mediated NINJ1 cleavage may represent an endogenous counter‐regulatory mechanism operating at sites of cardiovascular inflammation: by converting membrane‐bound NINJ1 to soluble sNINJ1, the system simultaneously removes a potential PMR executor (reducing the capacity for inflammatory cell death) while generating an anti‐inflammatory mediator (dampening ongoing inflammation). The balance between pro‐inflammatory full‐length NINJ1 and anti‐inflammatory sNINJ1 at inflammatory sites may be a critical determinant of whether inflammation resolves or persists. Additionally, muscimol—a GABA‐A receptor agonist—has been identified as a small‐molecule inhibitor of NINJ1 oligomerisation, with demonstrated preclinical efficacy in inflammatory disease models.^38^ Unlike glycine, which acts indirectly through modulation of membrane biophysical properties (see Section 5.2), muscimol appears to interfere more directly with NINJ1 assembly processes. Demonstration of therapeutic benefit in preclinical inflammatory disease models provides robust proof of concept for pharmacological targeting of NINJ1 and positions muscimol as a promising lead compound for NINJ1‐targeted drug development in cardiovascular applications.

A particularly important regulatory mechanism involves proteolytic cleavage by MMP‐9. MMP‐9 directly cleaves membrane‐bound NINJ1 to generate a soluble N‐terminal ectodomain fragment (sNINJ1).^39^ This soluble form exhibits distinct biological activities compared to membrane‐bound NINJ1, particularly in the context of cardiovascular inflammation. The sNINJ1 fragment contains the N‐NAM adhesion motif and exhibits chemotactic activity, as well as anti‐inflammatory properties.^9^

Importantly, NINJ1‐mediated PMR can be pharmacologically inhibited. Recent studies have identified glycine as a small molecule inhibitor that prevents NINJ1 oligomerisation by interfering with its membrane clustering.^40^ Additionally, monoclonal antibodies targeting NINJ1 have been developed that specifically block oligomerisation, effectively preventing PMR while preserving other cellular functions.^9^ These findings provide proof of concept for therapeutic targeting of NINJ1 in inflammatory diseases.

### 2.4 Cell‐death pathways and NINJ1: a critical and balanced appraisal

NINJ1 functions as a convergence point for multiple lytic cell‐death pathways, though its requirement varies significantly across cell types, death stimuli and cellular contexts. A nuanced understanding of these pathway‐specific relationships—including contentious areas where the field has not reached consensus—is essential for accurately evaluating NINJ1 as a therapeutic target in CVD. We present below a balanced appraisal of the current literature, clearly distinguishing established findings from areas of ongoing debate.

#### 2.4.1 Pyroptosis: NINJ1 requirement clearly established

Pyroptosis is a highly inflammatory form of programmed cell death initiated by pattern recognition receptor (PRR) activation—including NLRP3, AIM2, NLRC4 and pyrin inflammasomes responding to diverse pathogen‐associated molecular patterns and DAMPs—leading to inflammasome assembly, inflammatory caspase activation (primarily caspase‐1 via canonical pathway, caspase‐11/4/5 via non‐canonical pathway) and GSDMD cleavage.^41^ The released GSDMD N‐terminal domain oligomerises to form plasma membrane pores (∼18‒21 nm diameter) that permit selective efflux of mature IL‐1β (∼17 kDa), IL‐18 (∼18 kDa), potassium ions (driving further inflammasome activation) and ATP, while maintaining overall membrane integrity. NINJ1‐mediated PMR occurs downstream of GSDMD pore formation as a temporally and mechanistically distinct terminal event. The foundational study by Kayagaki et al. definitively demonstrated the essential role of NINJ1: NINJ1 knockout cells activated by multiple pyroptotic stimuli form GSDMD pores normally, release IL‐1β efficiently (confirming functional GSDMD pores), but critically fail to undergo PMR.^13^ Large intracellular proteins that cannot traverse GSDMD pores—including LDH (∼140 kDa) and HMGB1 (∼25 kDa)—are retained within NINJ1‐deficient cells rather than being released. Morphologically, NINJ1 knockout cells swell (due to osmotic water influx through GSDMD pores) but do not lyse, remaining in a ‘balloon‐like’ swollen state. This temporal separation—GSDMD pores forming within minutes of inflammasome activation, with NINJ1‐mediated rupture occurring minutes to hours later—may be therapeutically exploitable. Consensus: there is strong agreement that NINJ1 is required for PMR during pyroptosis. This is the best‐established and least controversial context for NINJ1 function.

#### 2.4.2 Necroptosis: context‐dependent and controversial

Necroptosis is a programmed form of necrotic cell death mediated by the receptor‐interacting protein kinase 1 (RIPK1)/RIPK3/MLKL signalling cascade, typically triggered when death receptor signalling (TNFR1, Fas, TRAIL‐R) or Toll‐like receptor signalling (TLR3 and TLR4) proceeds in the context of caspase‐8 inhibition or deficiency.^42^ RIPK3‐mediated phosphorylation of MLKL induces MLKL oligomerisation and translocation to the plasma membrane, where it causes membrane permeabilisation. The role of NINJ1 in necroptosis is more complex and context‐dependent than in pyroptosis, with conflicting evidence from methodologically rigorous studies. Evidence supporting NINJ1 involvement: Kayagaki et al. demonstrated that NINJ1 knockout partially protected against necroptotic membrane rupture in bone marrow‐derived macrophages,^13^ suggesting NINJ1 acts downstream of MLKL to execute final membrane disruption. Evidence questioning the absolute requirement for NINJ1: Dondelinger et al. demonstrated that MLKL possesses intrinsic membrane‐permeabilising activity and can directly induce membrane disruption in certain cell types.^26^ In HT‐29 cells (human colorectal adenocarcinoma), NINJ1 knockout did not prevent necroptotic cell death—membrane rupture occurred with normal kinetics despite NINJ1 absence.^26^, ^43^ This indicates that MLKL‐mediated membrane permeabilisation can be sufficient for cell lysis, without NINJ1 contribution, in some cellular contexts. Interpretation: these discrepant findings likely reflect genuine biological variability rather than technical artefacts: (1) cell‐type specificity—different cells express different levels of NINJ1 and MLKL, and the relative contributions may vary accordingly; (2) MLKL pore‐forming capacity—MLKL can directly form membrane pores, and whether this is sufficient for complete lysis may depend on expression levels and cellular factors; (3) kinetic differences—MLKL‐mediated permeabilisation may be slower but eventually sufficient without NINJ1 acceleration; (4) redundancy—in high‐NINJ1 cells, NINJ1 may accelerate PMR, while in low‐NINJ1 cells or high‐MLKL cells, MLKL alone may be sufficient. Current consensus: NINJ1 contributes to necroptotic membrane rupture in many but not all cellular contexts. The requirement is cell‐type dependent and condition dependent, and therapeutic strategies based on NINJ1 inhibition may have variable efficacy in blocking necroptosis, depending on the tissue context.

#### 2.4.3 Secondary necrosis: NINJ1‐dependent post‐apoptotic lysis

Secondary necrosis (post‐apoptotic lysis) occurs when apoptotic cells fail to be cleared by efferocytosis—the phagocytic removal of dying cells by macrophages and other professional phagocytes. Under normal circumstances, apoptotic cells expose ‘eat me’ signals (PS, calreticulin) and are rapidly cleared before membrane integrity is lost, maintaining immunological silence without DAMP release or inflammatory consequences. When efferocytosis is impaired (due to phagocyte dysfunction, overwhelming apoptotic burden, or defective recognition signals), apoptotic cells eventually undergo delayed membrane rupture, releasing their contents and triggering inflammation. David et al. demonstrated that this post‐apoptotic membrane rupture is NINJ1‐dependent: NINJ1 knockout cells undergoing apoptosis failed to lyse even when efferocytosis was experimentally prevented for extended periods (24‒48 h).^12^ This positions NINJ1 as a universal PMR executor that operates even during apoptotic cell death when clearance mechanisms fail. Cardiovascular relevance: in large myocardial infarctions that generate a massive apoptotic cardiomyocyte burden, efferocytic capacity may be overwhelmed, and secondary necrosis of uncleared apoptotic cells may significantly amplify inflammatory responses beyond the initial ischaemic injury. Similarly, in advanced atherosclerotic plaques where efferocytosis is impaired (defective MerTK signalling, oxidised lipid‐mediated phagocyte dysfunction), secondary necrosis may contribute to necrotic core expansion and plaque destabilisation.

#### 2.4.4 Ferroptosis: highly controversial—balanced presentation of conflicting evidence

Ferroptosis is an iron‐dependent form of regulated cell death characterised by overwhelming lipid peroxidation, typically triggered by glutathione depletion (via system Xc‐inhibition, e.g., erastin) or direct GPX4 inhibition (e.g., RSL3, ML162).^44^ Morphological features include shrunken mitochondria with increased membrane density and reduced cristae. Ferroptosis is mechanistically distinct from apoptosis, necroptosis and pyroptosis—it is not blocked by caspase inhibitors, RIPK1/RIPK3 inhibitors or GSDMD deficiency. The role of NINJ1 in ferroptosis remains highly contentious, with directly conflicting evidence from well‐conducted studies. We present both positions without favouring either interpretation: evidence supporting NINJ1 involvement: Ramos et al. reported that NINJ1 knockout substantially reduced membrane rupture during ferroptosis induced by multiple triggers (RSL3, erastin, ML162) across several cell types.^14^ In NINJ1‐deficient cells, LDH release was decreased, and membrane integrity was preserved longer despite ongoing lipid peroxidation. These authors proposed that NINJ1 executes PMR downstream of the lipid peroxidation cascade, similar to its role downstream of GSDMD in pyroptosis. Evidence against NINJ1 involvement: Hirata et al. presented compelling evidence that ferroptotic cell death proceeds independently of NINJ1.^45^ In their experimental systems, NINJ1 knockout did not prevent ferroptotic membrane rupture—cells died with normal kinetics and membrane permeabilisation. These authors proposed that severe lipid peroxidation can directly destabilise membranes without requiring protein‐mediated PMR execution, through mechanisms such as membrane thinning, increased permeability and eventual physical failure of extensively peroxidised lipid bilayers. Critical analysis of discrepancy: these conflicting findings may reflect: (1) cell‐type differences—different cells express different NINJ1 levels and have different ferroptotic sensitivities; (2) inducer differences—different ferroptosis inducers (GPX4 inhibitors vs. system Xc‐inhibitors) may produce different kinetics and severity of lipid peroxidation; (3) severity threshold—mild lipid peroxidation may require NINJ1 for membrane rupture, while severe peroxidation may directly destabilise membranes independent of any protein; (4) methodological differences—knockout versus knockdown completeness, different cell‐death readouts and timing of measurements. Our position: the field has not reached consensus on the role of NINJ1 in ferroptosis. Both studies appear methodologically sound, and the discrepancy likely reflects genuine biological complexity. Whether NINJ1‐targeted therapies would affect ferroptotic cell death remains uncertain and likely context dependent.

#### 2.4.5 SIGLEC12: an alternative PMR executor

A major recent advance that fundamentally expands our understanding of PMR machinery is the identification by Noh et al. of SIGLEC12 (sialic acid‐binding immunoglobulin‐like lectin 12) as another executioner capable of mediating PMR independently of NINJ1.^46^ SIGLEC12 is an inhibitory receptor of the SIGLEC family, previously characterised for glycan recognition functions in immune cell biology. Noh et al. discovered that SIGLEC12 can oligomerise and insert into membranes in a manner that parallels—but is mechanistically distinct from—NINJ1. Critically, SIGLEC12 can mediate PMR in cells lacking NINJ1, demonstrating functional independence. In cells expressing both SIGLEC12 and NINJ1, the proteins may have redundant or complementary roles in executing membrane rupture. Implications of SIGLEC12 discovery: (1) complexity—the cellular machinery for PMR is more complex than ‘NINJ1 does everything’, involving multiple executioners with context‐specific roles; (2) redundancy—multiple PMR executors may provide backup mechanisms ensuring that inflammatory cell death can proceed through alternative pathways; (3) tissue specificity—expression patterns of NINJ1 versus SIGLEC12 differ across tissues and cell types, potentially determining which executor predominates in different pathological settings; (4) therapeutic implications—NINJ1 inhibition alone may not prevent PMR in cells that express functional SIGLEC12, suggesting that combination approaches targeting multiple executors may be required for complete PMR blockade in some contexts. Cardiovascular relevance: SIGLEC12 expression patterns in cardiovascular cell types (endothelial cells, cardiomyocytes, VSMCs, cardiac macrophages) remain incompletely characterised. If SIGLEC12 is expressed in key cardiovascular populations, it may provide an alternative PMR pathway that could limit the efficacy of NINJ1‐targeted monotherapies. Collective interpretation: NINJ1 is a major—and in many contexts essential—executor of PMR, but it is not universally required across all lytic death pathways and cell types. Alternative executioners, including direct MLKL‐mediated membrane disruption (in necroptosis), lipid peroxidation‐induced membrane failure (potentially in ferroptosis) and SIGLEC12‐dependent PMR, provide redundancy in this critical cellular process. Therapeutic strategies targeting NINJ1 must consider these nuances to predict efficacy across different disease contexts, cell populations and death stimuli.

### 2.5 Reconciling the N‐NAM paradox: adhesion functions in light of intracellular N‐terminus topology

The N‐NAM (residues Pro26‐Asn37) was originally identified and characterised by Araki and Milbrandt as the domain mediating NINJ1's homophilic cell‒cell adhesion properties.^7^ Their studies demonstrated that synthetic peptides corresponding to N‐NAM could inhibit NINJ1‐mediated cell aggregation when applied extracellularly, and antibodies targeting N‐NAM epitopes blocked adhesion functions. Subsequent studies extended these observations to vascular contexts, with N‐NAM‐containing peptides demonstrating chemotactic activity for endothelial cells^19^ and the N‐NAM‐containing sNINJ1 fragment exhibiting anti‐inflammatory effects on macrophages by suppressing NF‐κB and mitogen‐activated protein kinase (MAPK) signalling.^9^ However, the recent cryo‐EM structural determination by Pourmal et al., which definitively positions the N‐terminus—and therefore the N‐NAM motif—intracellularly in the autoinhibited NINJ1 homodimer, creates a fundamental paradox: how can a domain sequestered in the cytoplasm mediate extracellular adhesion functions?^16^ Critical evaluation of evidence strength: the structural data from Pourmal et al. are methodologically rigorous. Cryo‐EM provides direct visualisation of protein architecture in near‐native membrane environments, avoiding artefacts associated with earlier biochemical approaches (overexpression systems that may saturate membrane insertion machinery, antibody perturbation that may trap non‐native conformations, analysis of recombinant fragments that do not recapitulate full‐length membrane‐embedded protein behaviour). The structure is internally consistent with functional autoinhibition data—the sequestration of amphipathic helices explains why NINJ1 is inactive until death signals trigger conformational change. We find no compelling reasons to question this structural determination. The historical adhesion function data, while extensive and reproducible, derived predominantly from experimental systems that may not preserve native membrane topology: (1) studies used overexpressed NINJ1, which may adopt non‐native configurations when membrane insertion machinery is saturated; (2) antibody accessibility studies may have detected protein in non‐native conformations or during biosynthetic trafficking before final topology is achieved; (3) peptide competition experiments demonstrate that free N‐NAM peptides have biological activity, but this does not prove that the N‐NAM is extracellularly accessible in the context of full‐length membrane‐embedded NINJ1; (4) recombinant protein fragments behave differently from full‐length membrane‐embedded protein. Proposed reconciliation—sNINJ1 as the adhesion‐competent form: we propose that the most parsimonious reconciliation is that soluble sNINJ1, generated by MMP‐9‐mediated proteolytic cleavage between Leu56 and Leu57, represents the physiologically relevant adhesion‐competent form of NINJ1. Once the N‐terminal ectodomain is cleaved and liberated from membrane constraints, the N‐NAM motif is freed from topological restrictions and can engage in extracellular interactions with partner cells, matrix components or putative receptors. This interpretation is strongly supported by multiple observations: (1) recombinant sNINJ1 and synthetic N‐NAM peptides fully recapitulate the chemotactic, adhesion and anti‐inflammatory functions attributed to NINJ1 in the historical literature; (2) MMP‐9 is abundantly expressed at the inflammatory sites (atherosclerotic plaques, infarcted myocardium, aneurysmal tissue) where NINJ1 adhesion/signalling functions have been documented; (3) this model elegantly explains the dual‐role paradox that constitutes the central theme of this review: membrane‐bound full‐length NINJ1 executes pro‐inflammatory PMR, while cleaved soluble sNINJ1 mediates anti‐inflammatory signalling—a functional segregation based on proteolytic processing state. Alternative possibilities: While we favour the sNINJ1 hypothesis, alternative explanations cannot be definitively excluded: (1) NINJ1 may exist in multiple topological states in vivo, with the cryo‐EM structure capturing the predominant but not exclusive configuration; under specific cellular conditions, alternative conformations exposing the N‐terminus extracellularly might exist. (2) During the conformational transition from autoinhibited dimer to active filament, the N‐terminus reorients—brief extracellular exposure might occur during this dynamic process. (3) Historical experimental conditions may have captured biosynthetic intermediates or stress‐induced conformational variants not representative of the steady‐state membrane‐embedded protein. Resolution of this paradox will require: (1) live‐cell topology studies using membrane‐impermeant fluorescence probes or selective biotinylation to determine N‐terminus accessibility in intact cells under various conditions; (2) direct functional comparison of full‐length membrane‐bound NINJ1 versus cleaved sNINJ1 in adhesion and signalling assays; (3) studies in MMP‐9 knockout or inhibitor‐treated systems to assess NINJ1 functions in the absence of sNINJ1 generation; and (4) single‐molecule imaging to track NINJ1 conformational dynamics in living cells.

## 3 NINJ1 IN VASCULAR CELL BIOLOGY

### 3.1 Endothelial cells and vascular homeostasis

Endothelial cells form the critical interface between blood and tissues, regulating vascular tone, permeability, leukocyte trafficking and thrombosis.^47^ NINJ1 expression in endothelial cells has been documented across multiple vascular beds, and its functions in these cells are multifaceted and context dependent.^48^, ^49^

In the healthy endothelium, NINJ1 contributes to vascular homeostasis through its adhesive properties. The N‐NAM domain mediates homophilic interactions that support endothelial cell‒cell contacts and barrier function.^19^, ^49^ However, under pathological conditions, NINJ1 expression is upregulated, and its functions shift towards promoting inflammation and dysfunction.^50^

**FIGURE 3:**
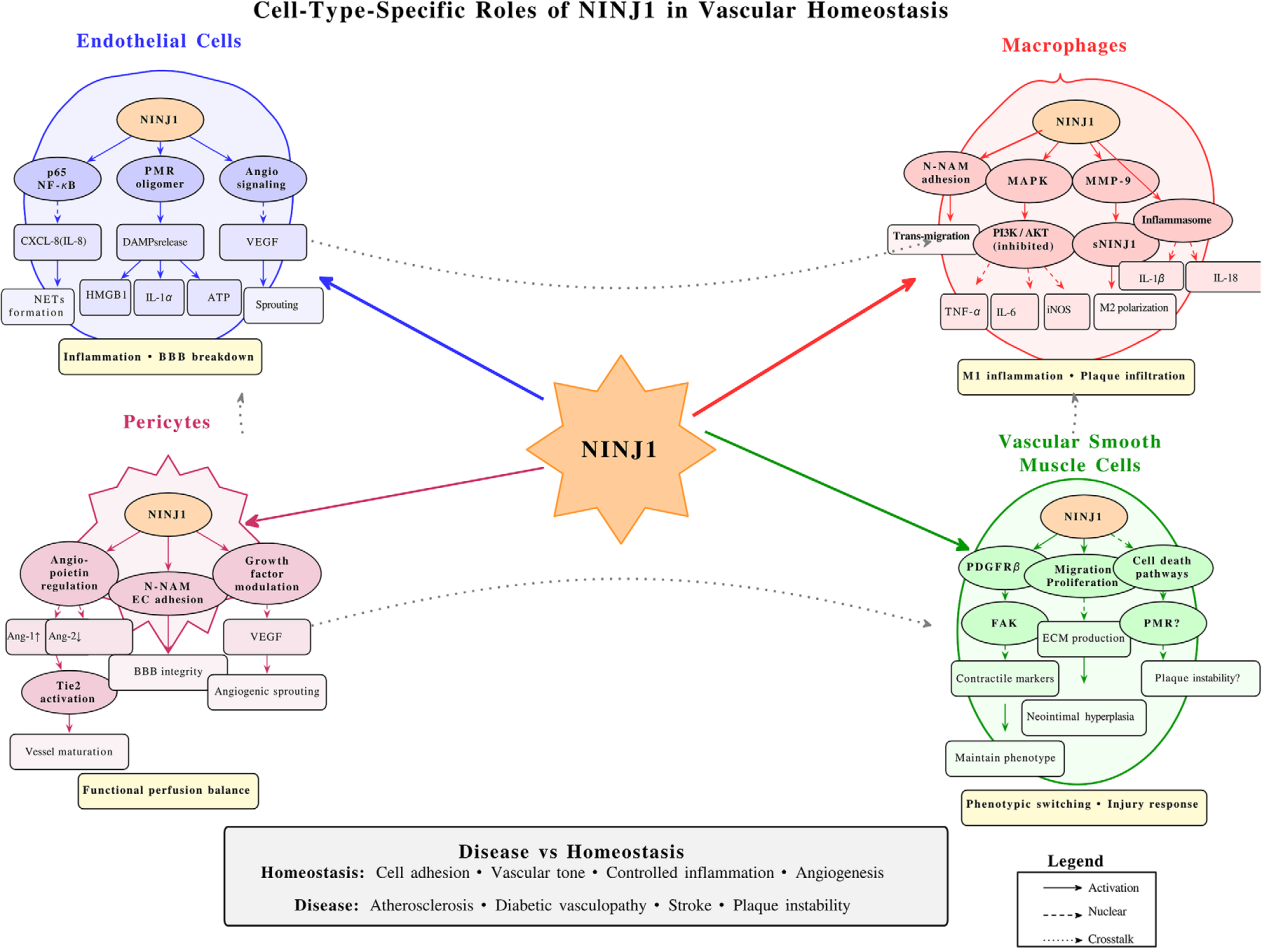
Cell‐Type‐Specific Roles of NINJ1 in Vascular Homeostasis and Disease. NINJ1 orchestrates distinct signaling pathways across major vascular cell types, regulating both homeostatic functions and pathological processes. In endothelial cells (blue), NINJ1 activates the NF‐κB/CXCL‐8 axis, promoting neutrophil recruitment and NET formation, mediates plasma membrane rupture (PMR) leading to DAMP release (HMGB1, IL‐1α, ATP), and modulates angiogenic signaling through VEGF, collectively contributing to inflammation, blood‐brain barrier breakdown, and atherosclerosis progression. In macrophages (red), NINJ1 facilitates trans‐endothelial migration via N‐NAM homophilic adhesion, promotes M1 polarization through MAPK activation and PI3K/Akt inhibition with subsequent expression of pro‐inflammatory mediators (TNF‐α, IL‐6, iNOS), undergoes MMP‐9‐mediated proteolytic cleavage to generate soluble NINJ1 (sNINJ1) that paradoxically promotes M2 polarization, and enables inflammasome‐dependent IL‐1β and IL‐18 release through PMR. In pericytes (purple), NINJ1 regulates the angiopoietin‐1/angiopoietin‐2 ratio to control Tie2 activation and vessel maturation, mediates N‐NAM‐dependent endothelial cell adhesion, maintaining blood‐brain barrier integrity, and modulates VEGF expression to balance angiogenic sprouting with functional perfusion. In vascular smooth muscle cells (green), NINJ1 influences PDGFRβ/FAK signaling to maintain the contractile phenotype, regulates migration, proliferation, and ECM production, thereby contributing to neointimal hyperplasia, and potentially mediates cell death through PMR, thereby contributing to plaque instability. Dotted arrows indicate intercellular crosstalk pathways. Under homeostatic conditions, NINJ1 maintains cell adhesion, vascular tone, and controlled inflammation, and promotes balanced angiogenesis. During disease states, including atherosclerosis, diabetic vasculopathy, stroke, and vascular injury, dysregulated NINJ1 expression and function drive pathological vascular remodeling and inflammation. Solid arrows, activation; dashed arrows, nuclear translocation or transcriptional regulation; dotted arrows, intercellular crosstalk.

Recent studies have revealed that NINJ1 drives atherosclerosis progression in endothelial cells (Figure 3) through activation of the NF‐κB/CXCL‐8 (IL‐8) signalling axis.^51^ In human umbilical vein endothelial cells (HUVECs) and atherosclerotic mouse models, NINJ1 overexpression or stimulation promotes nuclear translocation of p65 NF‐κB and increases expression of the pro‐inflammatory chemokine CXCL‐8.^51^, ^52^ This chemokine recruits neutrophils and promotes neutrophil extracellular trap (NET) formation, thereby exacerbating endothelial injury and atherosclerotic plaque instability.^53^

Diabetic vascular complications involve significant endothelial dysfunction, and NINJ1 has emerged as a key mediator of this pathology. Studies using high glucose‐treated endothelial cells demonstrate that NINJ1 upregulation contributes to endothelial injury and dysfunction through multiple mechanisms.^48^, ^49^ Functional blocking of NINJ1 using neutralising antibodies or peptide inhibitors protects endothelial cells from hyperglycaemia‐induced damage, reduces oxidative stress and preserves endothelial nitric oxide production.^54^ These findings suggest NINJ1 as a potential therapeutic target for diabetic vasculopathy.

The role of NINJ1 in endothelial cell death is particularly relevant in cardiovascular pathology. During inflammatory cell death, endothelial NINJ1 mediates PMR, releasing DAMPs (Figure 3), including HMGB1, IL‐1α and ATP.^12^, ^13^ It is crucial to distinguish the mechanistic and sequential functions of GSDMD and NINJ1 in cytokine and DAMP release during pyroptosis. Schachter et al. demonstrated, using genetically modified macrophages and real‐time biosensors, that ATP release is an early, transient danger signal mediated by GSDMD pores, occurring independently of NINJ1 and preceding both IL‐1β release and cell death.^55^ The mature IL‐1beta (17 kDa) and IL‐18 cytokines, which are cleaved by inflammasome‐activated caspase‐1, are released in a size‐ and charge‐dependent manner via GSDMD pores.^24^, ^56^ Subsequently, NINJ1 facilitates final membrane rupture, enabling the release of larger intracellular DAMPs, such as LDH (140 kDa as a tetramer) and HMGB1 (25 kDa), which are too large to pass through GSDMD pores. Recognising this mechanistic and temporal distinction has significant implications for therapeutic strategies.^49^ Interestingly, NINJ1's effects on angiogenesis appear complex and dose dependent. In vitro studies using co‐culture systems of endothelial cells and pericytes suggest that moderate NINJ1 expression supports angiogenic sprouting, while high‐level expression may be inhibitory.^26^, ^40^ This biphasic effect may reflect NINJ1's dual roles in promoting cell‒cell interactions at physiological levels while triggering cell‐death pathways when overexpressed under pathological conditions.

### 3.2 Macrophages and inflammatory responses

Macrophages are central orchestrators of cardiovascular inflammation, and NINJ1 profoundly influences macrophage function in both homeostatic and pathological contexts.^57^ NINJ1 is highly expressed in macrophages and monocytes, particularly during inflammatory activation.^12^

One of the best‐characterised functions of NINJ1 in macrophages is transmigration and tissue infiltration. NINJ1 enhances the basal motility of immune cells (Figure 3), by inducing protrusive membrane dynamics.^58^ Through its N‐NAM adhesion motif, NINJ1 mediates interactions between macrophages and endothelial cells, facilitating transendothelial migration into inflamed tissues.^19^ In atherosclerosis models, NINJ1 deficiency in bone marrow‐derived cells reduces macrophage accumulation in atherosclerotic plaques, demonstrating its importance in lesion development.^12^, ^13^

Paradoxically, total NINJ1 deficiency in macrophages leads to enhanced inflammatory activation. Studies show that NINJ1‐deficient macrophages (Figure 3) exhibit elevated expression of pro‐inflammatory genes, including TNF‐α, IL‐6 and inducible nitric oxide synthase (iNOS).^12^, ^14^ This occurs through dysregulation of signalling pathways: NINJ1 deficiency enhances MAPK activation while inhibiting the phosphoinositide 3‐kinase/Akt (Figure 3b), pathway, skewing macrophages towards a pro‐inflammatory M1 phenotype.^9^

The key to understanding this paradox lies in the proteolytic processing of NINJ1. Macrophages produce high levels of MMP‐9, which cleaves membrane‐bound NINJ1 to generate sNINJ1.^39^ This soluble form acts as an anti‐inflammatory mediator, reducing expression of pro‐inflammatory genes in both human and mouse macrophages.^59^ The sNINJ1 fragment exhibits chemotactic activity and can modulate macrophage polarisation towards an anti‐inflammatory M2‐like phenotype.^57^

This discovery has led to the development of sNINJ1‐mimetic peptides, including ML56 and PN12, which contain the N‐NAM sequence.^19^ These peptides recapitulate the anti‐inflammatory effects of natural sNINJ1, reducing pro‐inflammatory gene expression and attenuating monocyte transendothelial migration in vitro. In vivo studies demonstrate that continuous administration of these peptides reduces atherosclerosis in apolipoprotein E‐deficient mice, even in the complete absence of endogenous NINJ1, supporting their potential as therapeutic candidates warranting further preclinical development.^9^

MicroRNA‐125a‐5p has been identified as a post‐transcriptional regulator of NINJ1 in macrophages.^60^ This microRNA directly targets the 3′‐untranslated region of NINJ1 mRNA, thereby suppressing its expression. Overexpression of miR‐125a‐5p in macrophages reduces NINJ1 levels and attenuates macrophage‐mediated vascular dysfunction in diabetic retinopathy models.^60^ This regulatory axis offers additional therapeutic opportunities to modulate NINJ1‐dependent macrophage inflammation.

Recent studies on NINJ1‐mediated PMR in macrophages have revealed its critical role in DAMP release during inflammatory responses. Mature IL‐1β and IL‐18, processed by caspase‐1 during inflammasome activation, are released primarily through GSDMD pores prior to complete membrane rupture. NINJ1's role is to execute terminal PMR, enabling release of larger DAMPs (HMGB1, LDH and cytosolic proteins) that cannot pass through the smaller GSDMD pore channel.^13^, ^61^

#### 3.2.1 sNINJ1 generation: addressing MMP‐9 cleavage site accessibility

The MMP‐9 cleavage site between Leu56 and Leu57 resides within the α1 helical region, raising questions about accessibility given the structural constraints of the autoinhibited dimer.^39^ Several mechanisms may enable MMP‐9 access: (1) conformational dynamics—the autoinhibited dimer likely undergoes transient ‘breathing’ motions that transiently expose the cleavage site; protein structures represent energy minima, but thermal fluctuations sample alternative conformations. (2) Local unfolding at the membrane‒water interface—the junction between membrane‐embedded and aqueous regions may have increased conformational flexibility, permitting MMP‐9 access. (3) Processing during biosynthesis—cleavage may occur during trafficking before the final autoinhibited structure is achieved. (4) Alternative conformational states—stress conditions may favour conformations with exposed cleavage sites. The consistent detection of sNINJ1 in biological fluids confirms that cleavage occurs physiologically, despite structural considerations.

sNINJ1 exerts anti‐inflammatory effects through multiple mechanism^9^: (1) competitive inhibition/decoy function—sNINJ1 may compete with membrane‐bound NINJ1 for binding partners, reducing full‐length NINJ1 activity; (2) direct anti‐inflammatory signalling—sNINJ1 suppresses NF‐κB nuclear translocation and MAPK phosphorylation (ERK1/2, p38, JNK) in macrophages, reducing inflammatory gene transcription^9^; (3) macrophage polarisation—sNINJ1 promotes M2‐like anti‐inflammatory phenotype (increased CD206, Arg1 and IL‐10; decreased iNOS, TNF‐α and IL‐6); (4) chemotactic activity—the N‐NAM motif within sNINJ1 has documented chemotactic properties that may recruit repair‐associated cell populations.

Critically, the receptor mediating sNINJ1's biological effects remains unidentified—this represents a major knowledge gap in the field that significantly limits mechanistic understanding and therapeutic development. The rapid, specific signalling responses induced by sNINJ1 (NF‐κB suppression within minutes of exposure) suggest receptor‐mediated signalling rather than non‐specific effects. Candidate receptor classes include: (1) integrins—given N‐NAM's adhesion motif characteristics and RGD‐independent integrin interactions documented for related proteins; (2) PRRs—given sNINJ1's immunomodulatory effects resembling those of endogenous PRR modulators; (3) novel specific receptor—an uncharacterised receptor with specific sNINJ1 affinity. Identification of the sNINJ1 receptor through unbiased approaches (proximity labelling, cross‐linking mass spectrometry and CRISPR screens) would substantially advance therapeutic development by enabling rational design of sNINJ1 mimetics and receptor‐targeted interventions.

### 3.3 Vascular smooth muscle cells

VSMCs undergo phenotypic switching from a contractile to a synthetic state during vascular injury and atherosclerosis—a process central to neointimal hyperplasia and plaque formation.^62^ While NINJ1's role in VSMCs (Figure 3) has been less extensively studied than in endothelial cells or macrophages, emerging evidence suggests important regulatory functions.

NINJ1 expression has been detected in both human and mouse VSMCs, with expression levels modulated by inflammatory stimuli and growth factors.^63^ Studies in multiple cell types have demonstrated that NINJ1‐mediated PMR is required for the release of high‐molecular‐weight DAMPs.^12^, ^13^ However, the precise contribution of NINJ1‐dependent DAMP release to cardiovascular inflammation in vivo remains to be directly tested in cardiac‐specific models.

The role of the related family member NINJ2 in VSMC biology has been more extensively characterised. Recent studies demonstrate that NINJ2 regulates VSMC phenotypic switching through interaction with platelet‐derived growth factor receptor‐β (PDGFRβ) and subsequent modulation of focal adhesion kinase signalling.^64^ NINJ2 deficiency promotes PDGF‐BB‐mediated VSMC dedifferentiation, proliferation and migration, while NINJ2 overexpression maintains the contractile phenotype.^64^, ^65^ Given the structural and functional similarities between NINJ1 and NINJ2, analogous regulatory mechanisms may exist in VSMCs for NINJ1, although this remains to be experimentally validated.

#### 3.3.1 NINJ1 and NINJ2: structural parallelism and functional divergence

While NINJ1 and its homologue NINJ2 share approximately 55% amino acid sequence identity and conserved two‐pass transmembrane architecture, they exhibit fundamentally distinct functions that preclude extrapolation of findings between paralogues—a critical point that must be emphasised given the historical conflation of these proteins in some literature. Most critically, NINJ2 cannot execute PMR. This functional divergence was definitively demonstrated by Sahoo et al., who showed that NINJ2 completely fails to induce membrane rupture despite its structural similarity to NINJ1.^11^ Through systematic mutagenesis and chimeric protein analysis, they identified specific residues within the amphipathic α‐helices of NINJ1—including key hydrophobic amino acids at positions that contact membrane lipids—that confer unique membrane‐destabilising properties absent in NINJ2. When these NINJ1‐specific residues were substituted with NINJ2 sequences, PMR activity was abolished; conversely, introducing NINJ1‐specific residues into NINJ2 partially restored PMR activity. This molecular dissection explains why NINJ2 cannot substitute for NINJ1 in lytic cell death and why NINJ1‐targeted therapies would not be compensated by NINJ2 upregulation. In VSMCs, NINJ2—but notably not NINJ1—has been characterised as a regulator of phenotypic switching through interaction with PDGFRβ signalling.^64^ NINJ2 modulates VSMC transitions between contractile and synthetic phenotypes, which are relevant to neointima formation and vascular remodelling. These NINJ2‐specific functions in VSMCs should not be extrapolated to NINJ1, which likely has distinct roles in VSMCs related to inflammatory cell death and DAMP release rather than phenotypic regulation. GWAS have linked polymorphisms near the NINJ2 locus, rather than the NINJ1 locus, with ischaemic stroke risk.^66^ These genetic associations are specific to NINJ2 and should not be interpreted as evidence for NINJ1 involvement in stroke pathogenesis. The mechanistic basis of NINJ2‒stroke associations remains unclear but may relate to NINJ2's roles in vascular cell biology rather than inflammatory cell death. This review focuses specifically on NINJ1 and its unique function as a PMR executor. Findings regarding NINJ2 are included only where they illuminate NINJ1 biology through contrast, not conflation.

### 3.4 Pericytes and angiogenesis

Pericytes are mural cells that surround capillaries and small vessels and play critical roles in vascular development, maturation and stability.^67^ NINJ1 has emerged as an important regulator of pericyte function (Figure 3), particularly in angiogenesis and vascular repair.^63^, ^68^

NINJ1 expression is markedly upregulated in capillary pericytes during angiogenesis.^68^ Microarray analysis of pericytes co‐cultured with aortic tissue in three‐dimensional Matrigel systems identified NINJ1 as a differentially expressed gene associated with neovascularisation.^69^ Subsequent studies confirmed that NINJ1 expression in pericytes is further enhanced by hypoxia and inflammatory cytokines such as TNF‐α, suggesting its involvement in pathological angiogenesis.^52^, ^68^

Interestingly, the functional effects of NINJ1 in pericytes appear counterintuitive. Small interfering RNA‐mediated knockdown of NINJ1 in pericytes^3^ enhances their angiogenic effects, increasing endothelial tube formation and sprouting from aortic explants.^63^, ^70^ Conversely, NINJ1 overexpression in pericytes attenuates angiogenesis.^70^ This negative regulatory role appears to be mediated through modulation of angiogenic growth factor production. NINJ1 downregulation in pericytes increases expression of vascular endothelial growth factor and angiopoietin‐1 (Ang‐1), while reducing angiopoietin‐2 (Ang‐2) (Figure 3), thereby promoting a pro‐angiogenic environment.^68^, ^70^

However, the in vivo role of pericyte NINJ1 is more complex. Studies using pericyte‐specific NINJ1 knockout mice (NG2‐CreERT/Ninj1‐flox) subjected to hindlimb ischaemia demonstrate that pericyte NINJ1 is actually required for proper vessel maturation and functional blood flow recovery.^70^, ^71^ Pericyte‐specific NINJ1 deletion results in increased total microvessel density but reduced functional perfusion, indicating formation of immature, non‐functional vessels.^70^ This apparent contradiction with in vitro findings likely reflects NINJ1's dual roles: while its downregulation may promote initial angiogenic sprouting, sustained NINJ1 expression is necessary for proper pericyte‒endothelial interactions and vessel maturation.

Mechanistically, pericyte NINJ1 regulates the expression of angiopoietins, which are critical for pericyte recruitment and vessel stabilisation.^68^, ^70^ NINJ1 overexpression enhances Ang‐1 expression while suppressing Ang‐2, favouring vessel maturation. The Ang‐1/Ang‐2 ratio is a key determinant of vascular stability, with Ang‐1 promoting pericyte recruitment and vessel maturation through Tie2 receptor activation (Figure 3) on endothelial cells.^18^

These findings position pericyte NINJ1 as a critical regulator of the balance between angiogenic sprouting and vessel maturation—a balance that is often disrupted in cardiovascular pathologies. In ischaemic heart disease and peripheral arterial disease, therapeutic angiogenesis strategies must promote not only new vessel formation but also the development of functional, mature vasculature capable of sustained perfusion. Understanding and potentially manipulating pericyte NINJ1 may offer approaches to optimise therapeutic angiogenesis.

## 4 NINJ1 IN CARDIOVASCULAR PATHOLOGIES

### 4.1 Atherosclerosis and coronary artery disease

Atherosclerosis, the underlying pathology of most CVD, involves chronic inflammation of the arterial wall with accumulation of lipids, immune cells and extracellular matrix.^55^ NINJ1 plays multifaceted and sometimes opposing roles in atherogenesis, depending on its form (membrane‐bound vs. soluble) and cellular context.^9^, ^68^

#### 4.1.1 Clinical evidence

Clinical studies have established the relevance of NINJ1 in human atherosclerotic disease. Elevated serum levels of NINJ1 correlate with increased risk of large artery atherosclerotic acute ischaemic stroke.^72^ Analysis of atherosclerotic tissue from patients with coronary artery disease reveals increased NINJ1 expression compared to healthy arterial tissue.^9^ These findings suggest that NINJ1 may serve as a biomarker for atherosclerotic burden and cardiovascular risk.

#### 4.1.2 Membrane‐bound NINJ1 and atherosclerosis promotion

Genetic deletion studies have provided insights into the role of membrane‐bound NINJ1 in atherosclerosis. Paradoxically, both whole‐body NINJ1 knockout mice (Ninj1^−^/^−^Apoe^−^/^−^) and bone marrow‐specific NINJ1 deletion in atherosclerosis‐prone mice show exacerbated atherosclerotic lesion formation.^12^, ^23^ This worsening occurs through enhanced macrophage‐mediated inflammation. NINJ1‐deficient macrophages exhibit increased expression of pro‐inflammatory cytokines and chemokines, promoting greater monocyte recruitment and macrophage accumulation in plaques.^12^, ^73^

**FIGURE 4:**
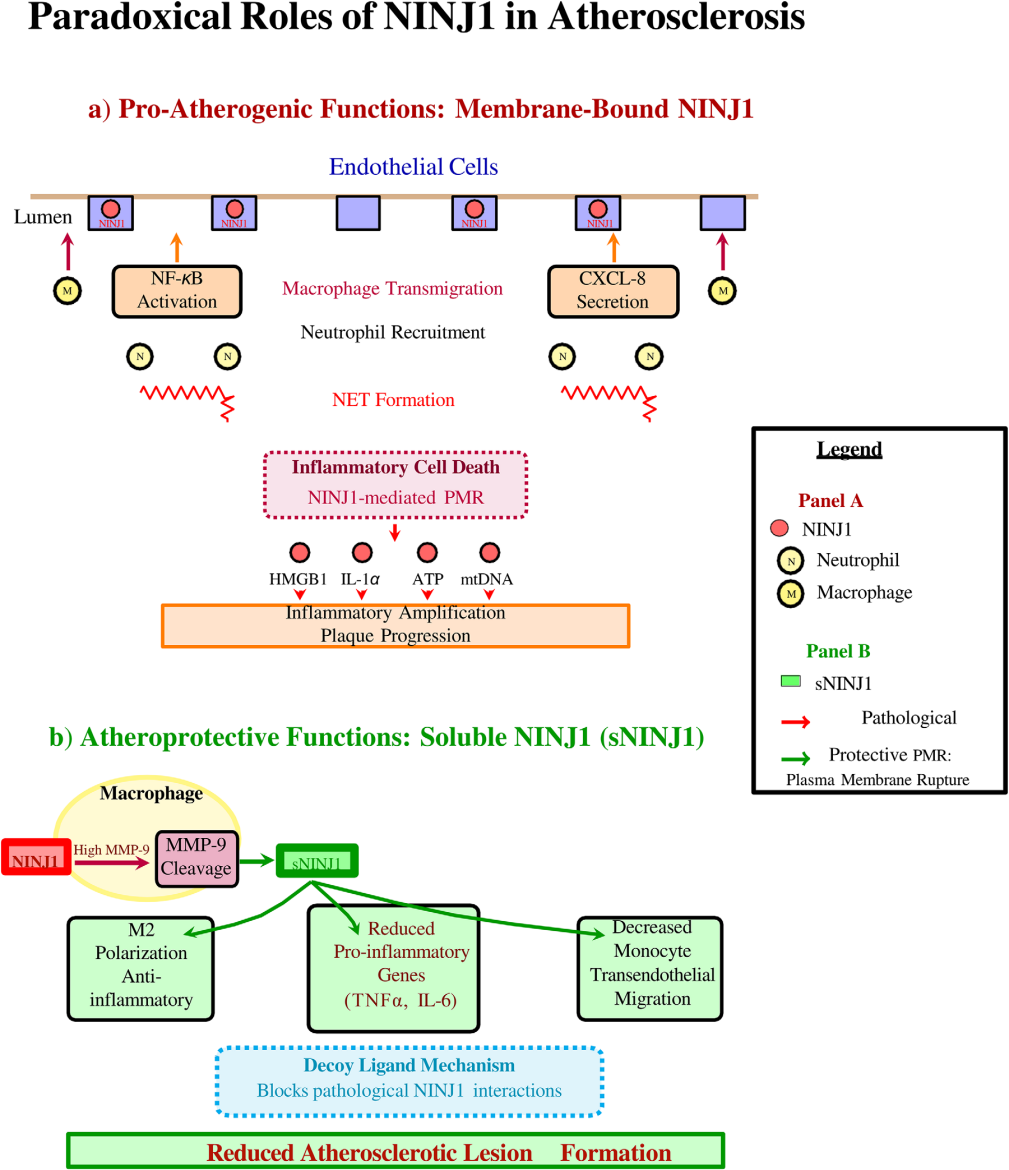
Paradoxical roles of NINJ1 in atherosclerosis: Pro‐atherogenic versus atheroprotective functions. (A) *Pro‐atherogenic functions of membrane‐bound NINJ1*: In endothelial cells, NINJ1 activates the NF‐κB/CXCL‐8 signaling axis, recruiting neutrophils and promoting neutrophil extracellular trap (NET) formation. NINJ1 facilitates macrophage transmigration into the subendothelial space. During inflammatory cell death, NINJ1‐mediated plasma membrane rupture (PMR) releases damage‐associated molecular patterns (DAMPs), including HMGB1, IL‐1α, ATP, and mitochondrial DNA, which activate surrounding cells and create inflammatory amplification loops that drive plaque progression. (B) *Atheroprotective functions of soluble NINJ1*: Matrix metalloproteinase‐9 (MMP‐9), abundantly expressed in plaque macrophages, cleaves membrane‐bound NINJ1 to generate soluble NINJ1 (sNINJ1). his soluble form promotes M2‐like macrophage polarization, suppresses pro‐inflammatory gene expression (TNF‐$∖alpha$, IL‐6), and reduces monocyte transendothelial migration. sNINJ1 acts as a decoy ligand, preventing pathological NINJ1‐mediated interactions and reducing atherosclerotic lesion formation. Paradoxical roles of Ninjurin1 (NINJ1) in atherosclerosis.

More recent work has elucidated additional pro‐atherogenic functions of NINJ1 in endothelial cells. Sun et al. demonstrated that NINJ1 silencing in HUVECs suppressed NF‐κB signalling and reduced expression of the pro‐inflammatory chemokine CXCL‐8 (IL‐8), conferring protection against oxidised LDL(low‐density lipoprotein)‐induced endothelial dysfunction by enhancing cell proliferation and migration while reducing apoptosis (Figure 4a).^51^ In ApoE^−/−^ mice, pharmacological NINJ1 inhibition with the mPN12 peptide significantly attenuated atherosclerotic plaque development and lipid accumulation while preserving collagen content.^48^ While CXCL‐8 is a well‐characterised neutrophil chemoattractant and has been implicated in NET formation in separate studies, direct evidence linking NINJ1‐mediated CXCL‐8 expression to NET‐dependent plaque destabilisation remains lacking. Nonetheless, these findings establish NINJ1 as a novel activator of the NF‐κB/CXCL‐8 axis in endothelial cells, highlighting its causal role in atherogenesis.^51^

#### 4.1.3 Soluble NINJ1 and atheroprotection

The breakthrough discovery that sNINJ1 exhibits anti‐inflammatory and atheroprotective effects has fundamentally altered our understanding of NINJ1's role in atherosclerosis.^9^ MMP‐9, which is abundantly expressed in macrophages within atherosclerotic plaques, cleaves membrane‐bound NINJ1 to generate sNINJ1 (Figure 4b).^39^ This soluble form acts as a decoy or signalling molecule that suppresses macrophage activation.

In vitro studies demonstrate that recombinant sNINJ1 or sNINJ1‐mimetic peptides (containing the N‐NAM sequence) reduce expression of pro‐inflammatory genes in classically activated (M1) macrophages.^19^ These effects involve modulation of multiple signalling pathways, including suppression of NF‐κB and MAPK activation.^74^ Importantly, sNINJ1 also reduces monocyte transendothelial migration, potentially limiting immune cell infiltration into developing plaques.^19^

In vivo validation comes from studies using sNINJ1‐mimetic peptides. Continuous subcutaneous infusion of the peptide mPN12 significantly reduces atherosclerotic lesion area in both Apoe^−^/^−^ and Ldlr^−^/^−^ mice fed high‐fat diets.^9^ Remarkably, these atheroprotective effects persist even in Ninj1^−^/^−^ mice, demonstrating that the peptides act through NINJ1‐independent mechanisms, possibly by modulating downstream inflammatory pathways.^9^, ^61^ This finding has important therapeutic implications, suggesting that sNINJ1 mimetics could benefit patients regardless of their endogenous NINJ1 status.

#### 4.1.4 Therapeutic implications

The dual nature of NINJ1 in atherosclerosis—providing protective effects through its soluble form (sNINJ1) while driving inflammation through its membrane‐bound form that mediates PMR—creates a complex therapeutic landscape in which indiscriminate inhibition could be harmful. Broadly blocking NINJ1 is expected to exacerbate atherosclerosis by eliminating sNINJ1's anti‐inflammatory signalling and simultaneously promoting a more pro‐inflammatory macrophage phenotype. Therefore, therapeutic strategies must be designed with precision. One approach involves promoting sNINJ1 generation by enhancing MMP‐9‐mediated cleavage of full‐length NINJ1 specifically within macrophages, thereby boosting the release of its beneficial soluble fragment; however, this requires careful control because MMP‐9 also degrades components of the extracellular matrix and has been linked to plaque destabilisation and increased risk of rupture.^9^, ^39^ A second strategy is the administration of sNINJ1 mimetics—engineered peptides, recombinant fragments, or stabilised protein analogs that reproduce the anti‐inflammatory functional domain of endogenous sNINJ1—an avenue that allows fine tuning of pharmacokinetics, tissue targeting, and resistance to proteolysis, all of which are areas of active research aimed at maximising efficacy.^9^, ^12^ A third, more selective strategy focuses on inhibiting NINJ1‐driven PMR without affecting the generation or function of sNINJ1; it targets NINJ1 oligomerisation and clustering that occur during pyroptosis or other inflammatory cell‐death pathways. By preventing this pore‐forming assembly—potentially through monoclonal antibodies that bind to oligomerisation interfaces or small molecules that disrupt conformational transitions—therapies could limit the release of DAMPs and thereby reduce inflammatory amplification while preserving NINJ1's beneficial roles.^14^, ^75^

### 4.2 Myocardial ischaemia‒reperfusion injury

IRI occurs when blood flow returns to previously ischaemic tissue, paradoxically causing additional damage through oxidative stress, calcium overload and inflammatory responses.^76^, ^77^ The role of NINJ1 in cardiac IRI can be inferred from mechanistic principles established in other contexts, though direct experimental evidence in the heart remains limited. Kayagaki et al. demonstrated that the anti‐NINJ1 monoclonal antibody D1 substantially protected against hepatic IRI in murine models, reducing hepatocellular necrosis, serum transaminase elevations and systemic DAMP release—providing proof‐of‐concept that NINJ1 inhibition can attenuate IRI in solid organs.^23^ Whether these findings directly translate to the myocardium requires further investigation, as cardiomyocytes differ substantially from hepatocytes in metabolism, apoptotic signalling and NINJ1 expression levels. The following discussion presents mechanistic hypotheses based on established NINJ1 biology that warrant experimental validation in cardiac‐specific models, rather than established facts.

#### 4.2.1 Mechanisms of NINJ1 in cardiac IRI

**FIGURE 5:**
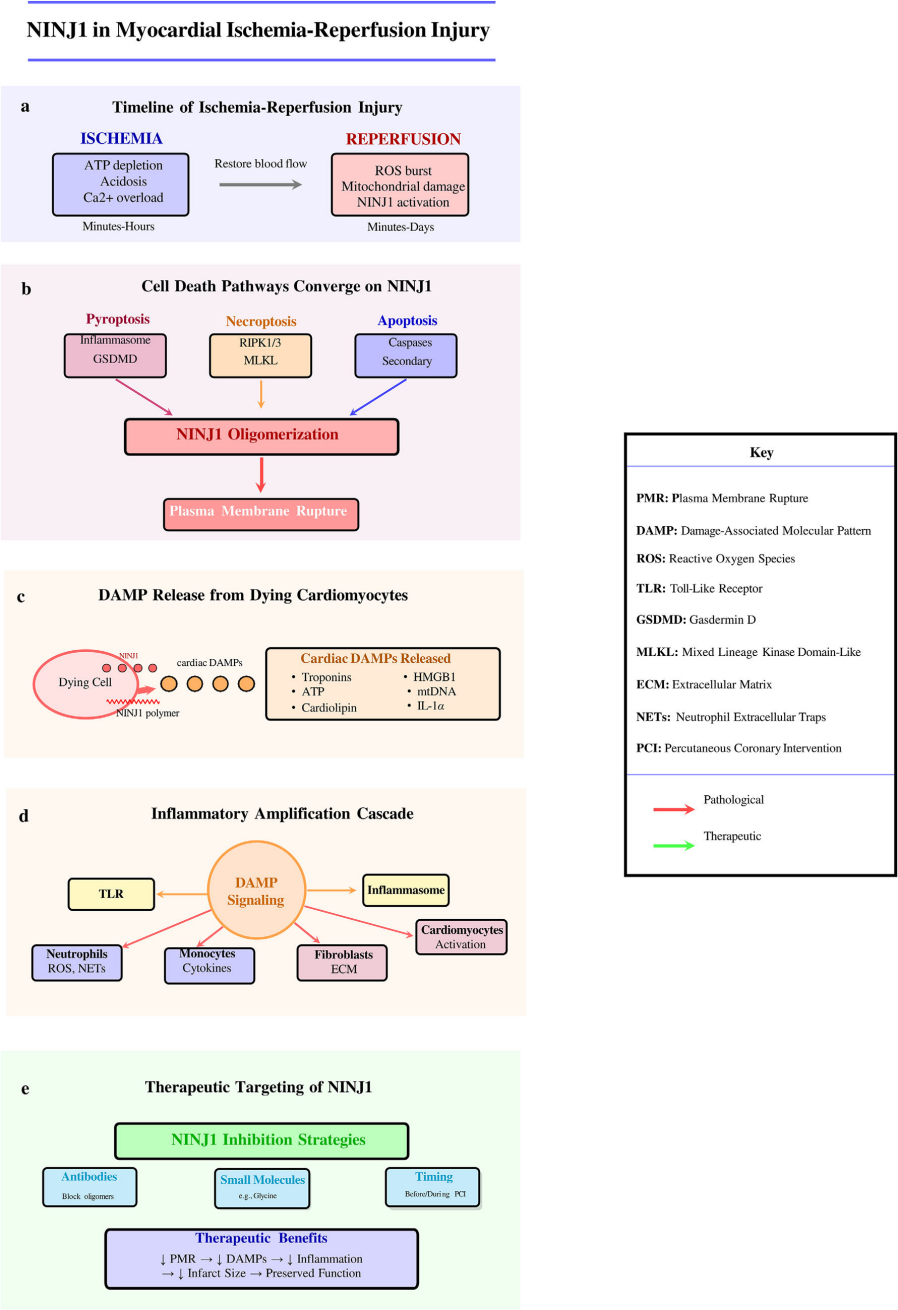
NINJ1‐mediated plasma membrane rupture in myocardial ischemia‐reperfusion injury. (A) Timeline of ischemia‐reperfusion injury: During ischemia (minutes to hours), ATP depletion, acidosis, and calcium dysregulation initiate multiple cell death pathways. Upon reperfusion (minutes to days), reactive oxygen species (ROS) generation and mitochondrial dysfunction trigger NINJ1‐mediated plasma membrane rupture (PMR). (B) Cell death pathway convergence: Multiple lytic cell death pathways—pyroptosis (via inflammasome activation and gasdermin D pore formation), necroptosis (via RIPK1/3 and MLKL activation), and secondary apoptosis (following caspase activation and failed efferocytosis)—all converge on NINJ1 oligomerization as the final common pathway to execute plasma membrane rupture. (C) DAMP release from dying cardiomyocytes: NINJ1 oligomers assemble at the plasma membrane of dying cardiomyocytes, leading to membrane rupture and release of cardiac damage‐associated molecular patterns (DAMPs), including troponins, HMGB1, ATP, mitochondrial DNA (mtDNA), cardiolipin, and IL‐1α. (D) Inflammatory amplification cascade: Released DAMPs activate pattern recognition receptors, including Toll‐like receptors (TLRs) and inflammasomes, on immune cells and surviving cardiomyocytes, triggering inflammatory amplification. This leads to neutrophil recruitment with ROS production and NET formation, monocyte recruitment with pro‐inflammatory cytokine release, fibroblast activation with excessive extracellular matrix (ECM) deposition, and activation of surviving cardiomyocytes. These processes collectively drive infarct expansion and adverse cardiac remodeling, ultimately leading to heart failure. (E) Therapeutic targeting of NINJ1: NINJ1 inhibition represents a promising therapeutic strategy for limiting reperfusion injury. Approaches include monoclonal antibodies that block NINJ1 oligomerization, small molecules such as glycine that prevent membrane clustering, with optimal timing before or during percutaneous coronary intervention (PCI). Therapeutic benefits include reduced PMR, decreased DAMP release, attenuated inflammation, smaller infarct size, and preserved cardiac function. The flexible therapeutic window makes NINJ1 inhibition clinically feasible for the treatment of acute myocardial infarction.

During myocardial ischaemia, ATP depletion, acidosis and calcium dysregulation trigger multiple cell‐death pathways (Figure 5), including apoptosis, necroptosis and pyroptosis.^78^, ^79^ Upon reperfusion, oxidative stress and mitochondrial dysfunction further activate these pathways. NINJ1‐mediated PMR represents the terminal event in these lytic cell‐death processes, enabling release (Figure 5b) of intracellular contents.^13^

The cardiac response to ischaemia‒reperfusion involves complex cross‐talk between multiple cell types, with NINJ1 emerging as a key mediator of intercellular inflammatory communication. During reperfusion, stressed or dying cardiomyocytes undergo NINJ1‐mediated PMR, releasing DAMPs into the extracellular space. These DAMPs activate PRRs (TLR4, RAGE and P2×7) on infiltrating neutrophils and monocytes, triggering NF‐κB‐dependent inflammatory gene expression and further cytokine release (IL‐1β, TNF‐α and IL‐6). This creates a feed‐forward inflammatory amplification loop. NINJ1 upregulation in cardiac endothelial cells during IRI enhances leukocyte adhesion and transmigration through homophilic N‐NAM interactions. Endothelial NINJ1 also activates the NF‐κB/CXCL‐8 axis, promoting neutrophil recruitment and NET formation that exacerbates microvascular obstruction and the no‐reflow phenomenon. Recruited macrophages encountering the DAMP‐rich post‐ischaemic environment undergo inflammasome activation and GSDMD‐dependent pyroptosis. Subsequent NINJ1‐mediated PMR releases mature IL‐1β and additional DAMPs, perpetuating the inflammatory cascade and expanding the area of injury beyond the initial ischaemic zone. This multicellular cross‐talk paradigm suggests that NINJ1 inhibition could interrupt inflammatory amplification at multiple nodes simultaneously—reducing cardiomyocyte DAMP release, limiting endothelial‐mediated leukocyte recruitment, and preventing macrophage pyroptosis—potentially offering superior cardioprotection compared with single‐target approaches.

Cardiomyocyte death with NINJ1‐dependent PMR releases large amounts of DAMPs (Figure 5c), including HMGB1, ATP, mitochondrial DNA and cardiac proteins such as troponins.^13^, ^14^, ^80^ These DAMPs activate PRRs on immune cells and surviving cardiomyocytes (Figure 5d), triggering sterile inflammation through NF‐κB and inflammasome activation.^81^ The resulting inflammatory cascade recruits neutrophils and monocytes (Figure 5d), which further damage the myocardium by producing ROS, proteases and pro‐inflammatory cytokines.^65^, ^82^

#### 4.2.2 Experimental evidence

Studies in hepatic IRI models provide strong proof‐of‐concept for NINJ1's role in reperfusion injury.^72^, ^80^ Kayagaki et al. demonstrate that anti‐NINJ1 antibody (clone D1) treatment protects in hepatic IRI models.^23^ Specifically, mice receiving anti‐NINJ1 antibody showed significantly reduced hepatocellular necrosis (histological injury score reduced by 58%), lower serum transaminases (alanine aminotransferase [ALT] reduced from 2847 ± 423 to 892 ± 156 U/L), diminished inflammatory cytokine levels, and reduced circulating DAMPs, including HMGB1, compared to isotype control‐treated animals. Importantly, treatment with anti‐NINJ1 monoclonal antibodies that block oligomerisation provides similar protection, demonstrating therapeutic potential.^23^

While direct evidence in cardiac IRI models remains limited, the principles established in hepatic and other organ systems likely apply to the heart. Studies examining NINJ1 expression in post‐infarction myocardium show upregulation in the infarct border zone, suggesting active involvement in cardiac injury responses.^83^ Given that cardiomyocyte pyroptosis and necroptosis contribute significantly to infarct expansion, NINJ1‐mediated PMR likely plays a central role in determining final infarct size.

#### 4.2.3 Therapeutic opportunities in cardiac IRI

The therapeutic window for targeting IRI spans the entire interval from the onset of ischaemia through the reperfusion phase, making it highly relevant to clinical scenarios such as acute myocardial infarction, in which reperfusion is restored through primary percutaneous coronary intervention or thrombolysis. While these interventions successfully re‐establish coronary blood flow, they do not address the secondary wave of tissue damage triggered by inflammatory processes during reperfusion, which remains a major unmet need.^84^ In this context, inhibiting NINJ1‐mediated PMR (Figure 5e) offers several mechanistic advantages. First, it provides considerable temporal flexibility: because NINJ1‐dependent PMR occurs mainly during reperfusion, when cell‐death pathways converge on terminal membrane rupture, anti‐NINJ1 agents could be administered either before or at the time of reperfusion, allowing broad applicability across different clinical settings.^9^ Second, NINJ1 inhibition acts at the final execution step of lytic cell death, blocking membrane rupture without suppressing upstream apoptotic, necroptotic or pyroptotic signalling events that may serve beneficial cellular quality‐control roles.^75^ By preventing only the rupture phase, dying cells can still undergo structured dismantling and be cleared more efficiently by phagocytes, while the release of DAMPs and pro‐inflammatory cytokines is minimised. Third, reducing DAMP‐driven inflammatory amplification could meaningfully limit infarct expansion: protecting vulnerable cardiomyocytes in the peri‐infarct zone from secondary inflammatory injury could preserve myocardial contractility and improve long‐term left‐ventricular function.^75^, ^85^ Finally, combination strategies may offer synergistic benefits. Pairing NINJ1 inhibitors with upstream inflammasome inhibitors or complement‐blocking agents could simultaneously modulate multiple inflammatory checkpoints, thereby strengthening cardioprotection and addressing the multifactorial nature of reperfusion injury.^61^, ^75^

### 4.3 Heart failure and cardiac remodelling

Following myocardial infarction or chronic pressure overload, the heart undergoes pathological remodelling characterised by cardiomyocyte hypertrophy, fibrosis and chamber dilatation—processes that ultimately lead to heart failure.^86^ Direct evidence linking NINJ1 to heart failure pathogenesis and cardiac remodelling remains preliminary and largely inferential. The following discussion presents mechanistic hypotheses based on NINJ1's characterised functions in inflammatory cell death that may be relevant to heart failure biology, but these hypotheses require systematic experimental testing before clinical translation. We present this section to stimulate research rather than assert established connections.

Chronic low‐grade inflammation is increasingly recognised as a driver of adverse cardiac remodelling.^87^ Persistent activation of cardiac immune cells, particularly macrophages, promotes fibroblast activation and extracellular matrix deposition.^88^ If NINJ1‐mediated inflammatory signalling contributes to this chronic inflammation, as suggested by its roles in other tissues, then NINJ1 could represent a therapeutic target for preventing or slowing heart failure progression.

Additionally, cardiomyocyte death through various pathways continues during the chronic phase post‐infarction and in non‐ischaemic cardiomyopathies.^74^ NINJ1‐dependent PMR accompanying this ongoing cell death may perpetuate inflammatory activation, creating a vicious cycle of cell death, inflammation and progressive dysfunction. Longitudinal studies examining NINJ1 expression and activity during cardiac remodelling would help clarify its role in heart failure pathogenesis.

### 4.4 Cerebrovascular disease and stroke

Although this review focuses primarily on cardiac and vascular pathology, NINJ1's role in cerebrovascular disease warrants discussion given the intimate relationship between the cardiovascular and cerebrovascular systems. GWAS initially linked the NINJ2 gene locus with increased risk of ischaemic stroke, prompting investigation of NINJ1 in cerebrovascular pathology.^66^, ^89^

Ischaemic stroke involves many pathophysiological processes analogous to myocardial infarction, including excitotoxicity, oxidative stress, inflammation and cell death.^90^ NINJ1‐mediated PMR has been implicated in neuronal and glial cell death following experimental stroke, with NINJ1 expression upregulated in the ischaemic penumbra.^91^ Importantly, blood‒brain barrier disruption—a major complication of stroke that exacerbates brain injury—involves endothelial cell death and tight junction breakdown, processes potentially mediated by NINJ1.^49^

The hemorrhagic transformation of ischaemic stroke, where reperfusion leads to bleeding into infarcted brain tissue, may also involve NINJ1‐dependent endothelial injury. Studies in stroke models show that inhibiting inflammatory cell‐death pathways reduces haemorrhagic transformation risk.^49^ Whether NINJ1‐specific inhibition provides similar protection remains to be determined but represents an important therapeutic consideration.

### 4.5 Diabetic cardiovascular complications

Diabetes mellitus dramatically increases CVD risk through multiple mechanisms including accelerated atherosclerosis, endothelial dysfunction and diabetic cardiomyopathy.^77^ NINJ1 has emerged as a mediator of several diabetic vascular complications, with particularly strong evidence in diabetic retinopathy that may extend to other vascular beds.^77^, ^85^

#### 4.5.1 Hyperglycaemia and NINJ1 upregulation

Chronic hyperglycaemia upregulates NINJ1 expression in multiple cell types through mechanisms involving oxidative stress, advanced glycation end products (AGEs) and inflammatory cytokines.^50^ In endothelial cells exposed to high glucose, NINJ1 expression increases significantly, correlating with markers of endothelial dysfunction including reduced nitric oxide production and increased expression of adhesion molecules.^19^

The transcription factor NF‐κB, which is activated by multiple diabetes‐associated stimuli, regulates NINJ1 transcription.^92^, ^93^ This creates a feed‐forward loop: hyperglycaemia activates NF‐κB, increasing NINJ1 expression, which then further activates NF‐κB through its effects on inflammatory pathways, perpetuating endothelial dysfunction and vascular inflammation.^51^

#### 4.5.2 Diabetic retinopathy

Diabetic retinopathy involves retinal microvascular complications, including increased permeability, capillary dropout and pathological neovascularisation.^94^ NINJ1 expression is markedly elevated in the retinas of diabetic animal models and in vitreous samples from patients with proliferative diabetic retinopathy.^54^, ^94^, ^95^

MicroRNA‐125a‐5p, a negative regulator of NINJ1, is downregulated in diabetic retinopathy.^60^ This derepression allows increased NINJ1 expression in retinal macrophages and vascular cells. Overexpression of miR‐125a‐5p in diabetic models suppresses NINJ1, reduces retinal inflammation, and ameliorates vascular dysfunction.^60^ These findings identify the miR‐125a‐5p/NINJ1 axis as a potential therapeutic target.

#### 4.5.3 Diabetic cardiomyopathy

Diabetic cardiomyopathy, characterised by left ventricular dysfunction in the absence of coronary artery disease or hypertension, is characterised by cardiomyocyte injury, fibrosis and impaired cardiac metabolism.^9^, ^96^ While direct evidence linking NINJ1 to diabetic cardiomyopathy remains limited, several lines of reasoning suggest potential involvement.

First, chronic low‐grade inflammation and cardiomyocyte death characterise diabetic hearts.^97^ If NINJ1‐mediated PMR accompanies cardiomyocyte death in this context, it would release DAMPs that activate cardiac fibroblasts and immune cells, promoting fibrosis. Second, endothelial dysfunction and microvascular rarefaction are characteristic of diabetic cardiomyopathy.^98^ NINJ1‐dependent endothelial injury could contribute to this microvascular loss. Third, metabolic dysregulation in diabetes increases susceptibility to ferroptosis, a form of cell death recently linked to NINJ1.^14^, ^99^ Investigating NINJ1's role in diabetic cardiomyopathy represents an important future direction.

#### 4.5.4 NINJ1‐metabolic pathway interactions in diabetic cardiovascular complications

The metabolic derangements characteristic of diabetes—hyperglycaemia, dyslipidemia and insulin resistance—create a milieu that promotes NINJ1‐mediated vascular injury through multiple interconnected mechanisms. Hyperglycaemia‒NLRP3‒NINJ1 axis: elevated glucose levels activate the NLRP3 inflammasome in endothelial cells and macrophages through multiple mechanisms, including ROS generation, thioredoxin‐interacting protein (TXNIP) upregulation and AGE receptor signalling. NLRP3 activation leads to GSDMD pore formation followed by NINJ1‐mediated PMR, establishing a direct link between hyperglycaemia and inflammatory cell death. Studies demonstrate that high glucose treatment upregulates NINJ1 expression in endothelial cells, sensitising them to pyroptotic stimuli. AGE‒RAGE‒NINJ1 signalling: Advanced glycation end‐products accumulate in diabetic vasculature and activate RAGE signalling, which upregulates NINJ1 transcription through NF‐κB‐dependent mechanisms. This AGE‐mediated induction of NINJ1 may explain the enhanced susceptibility of diabetic vessels to inflammatory injury. Lipotoxicity and ferroptosis: diabetic dyslipidemia, characterised by elevated free fatty acids and modified lipoproteins, promotes lipotoxic stress in vascular cells. Saturated fatty acids induce endoplasmic reticulum stress and ferroptosis—an iron‐dependent cell‐death pathway recently linked to NINJ1‐mediated PMR.^14^ Oxidised LDL also activates inflammasome pathways, connecting dyslipidemia to NINJ1‐dependent inflammatory responses. Insulin resistance and macrophage polarisation: insulin resistance promotes M1 macrophage polarisation, characterised by enhanced inflammasome activation and increased NINJ1‐dependent DAMP release. This contributes to the chronic low‐grade inflammation underlying diabetic vascular disease. miR‐125a‐5p as metabolic‐inflammatory link: the microRNA miR‐125a‐5p, which directly targets NINJ1 mRNA, is downregulated in diabetic conditions, leading to NINJ1 overexpression and enhanced macrophage‐mediated vascular dysfunction in diabetic retinopathy models.^60^ These metabolic‒inflammatory interactions suggest that NINJ1 inhibition may be particularly beneficial in diabetic patients, where metabolic stress chronically primes the NINJ1‐dependent inflammatory machinery.

### 4.6 Pulmonary hypertension and right heart failure

Pulmonary arterial hypertension (PAH) is characterised by pulmonary vascular remodelling, including endothelial dysfunction, smooth muscle cell proliferation and perivascular inflammation.^100^ The role of NINJ1 in PAH has not been directly investigated to date, representing a significant knowledge gap. The following discussion represents mechanistic speculation extrapolated from NINJ1's characterised functions in vascular cells and inflammatory contexts to the unique pathobiology of the pulmonary vasculature. These hypotheses are provided to identify potential research directions rather than describe established pathophysiology.

Endothelial cell apoptosis and dysfunction initiate PAH pathogenesis, followed by the emergence of apoptosis‐resistant endothelial cells that contribute to obliterative vascular lesions.^101^ NINJ1‐mediated PMR during the initial phase of endothelial cell death could release pro‐proliferative and inflammatory mediators that drive subsequent remodelling. Additionally, given NINJ1's role in regulating angiogenesis through pericyte function, its dysregulation could contribute to the disturbed angiogenesis characteristic of PAH.^68^

The right ventricular failure that develops secondary to increased afterload in PAH involves similar pathophysiological processes to left heart failure, including cardiomyocyte death, fibrosis and inflammation.^102^ Whether targeting NINJ1 could limit right ventricular remodelling in PAH models warrants investigation.

### 4.7 Aortic aneurysm and dissection

Aortic diseases represent life‐threatening cardiovascular conditions characterised by progressive wall degradation, chronic inflammation and structural failure. Recent investigations have identified NINJ1 as a significant contributor to aortic pathology. In abdominal aortic aneurysm, NINJ1 expression is markedly upregulated in aneurysmal tissue compared to non‐aneurysmal aorta, with prominent localisation to VSMCs undergoing inflammatory death and infiltrating macrophages in the degenerating media.^103^ NINJ1‐mediated PMR releases DAMPs, including HMGB1 and IL‐1α, that activate resident and recruited immune cells, driving MMP expression (MMP‐2, MMP‐9 and MMP‐12) and activity. These MMPs degrade elastic fibres and collagen, the structural proteins maintaining aortic wall integrity. NINJ1‐deficient mice exhibited attenuated aneurysm formation, preserved elastic laminae and reduced wall inflammation. The mechanical strain‐dependent NINJ1 activation described by Zhu et al. is particularly relevant to aneurysm biology: as aneurysmal segments dilate, wall tension increases according to Laplace's law (wall tension = transmural pressure × radius), potentially triggering NINJ1 activation in medial cells.^27^ This creates a devastating feed‐forward loop in which mechanical strain triggers NINJ1, which in turn induces PMR and DAMP release, activates MMPs that degrade the matrix, leading to further wall weakening, additional dilation and escalating mechanical strain. In aortic dissection, NINJ1 contributes to the inflammatory environment promoting medial degeneration and dissection propagation.^104^ NINJ1‐mediated inflammatory cell death in the media weakens the structural integrity required to resist dissection extension. Therapeutically, NINJ1 inhibition may slow aneurysm progression by limiting DAMP‐mediated inflammation and MMP activation. Given the lack of effective medical therapies for aneurysm (current management relies on surveillance and surgical intervention), NINJ1‐targeted approaches warrant preclinical investigation.

## 5 THERAPEUTIC TARGETING OF NINJ1

The multifaceted roles of NINJ1 in cardiovascular pathology have stimulated interest in therapeutic targeting. However, the complexity of NINJ1 biology—particularly the opposing functions of membrane‐bound versus soluble forms—necessitates carefully designed intervention strategies.

### 5.1 Monoclonal antibodies

**FIGURE 6:**
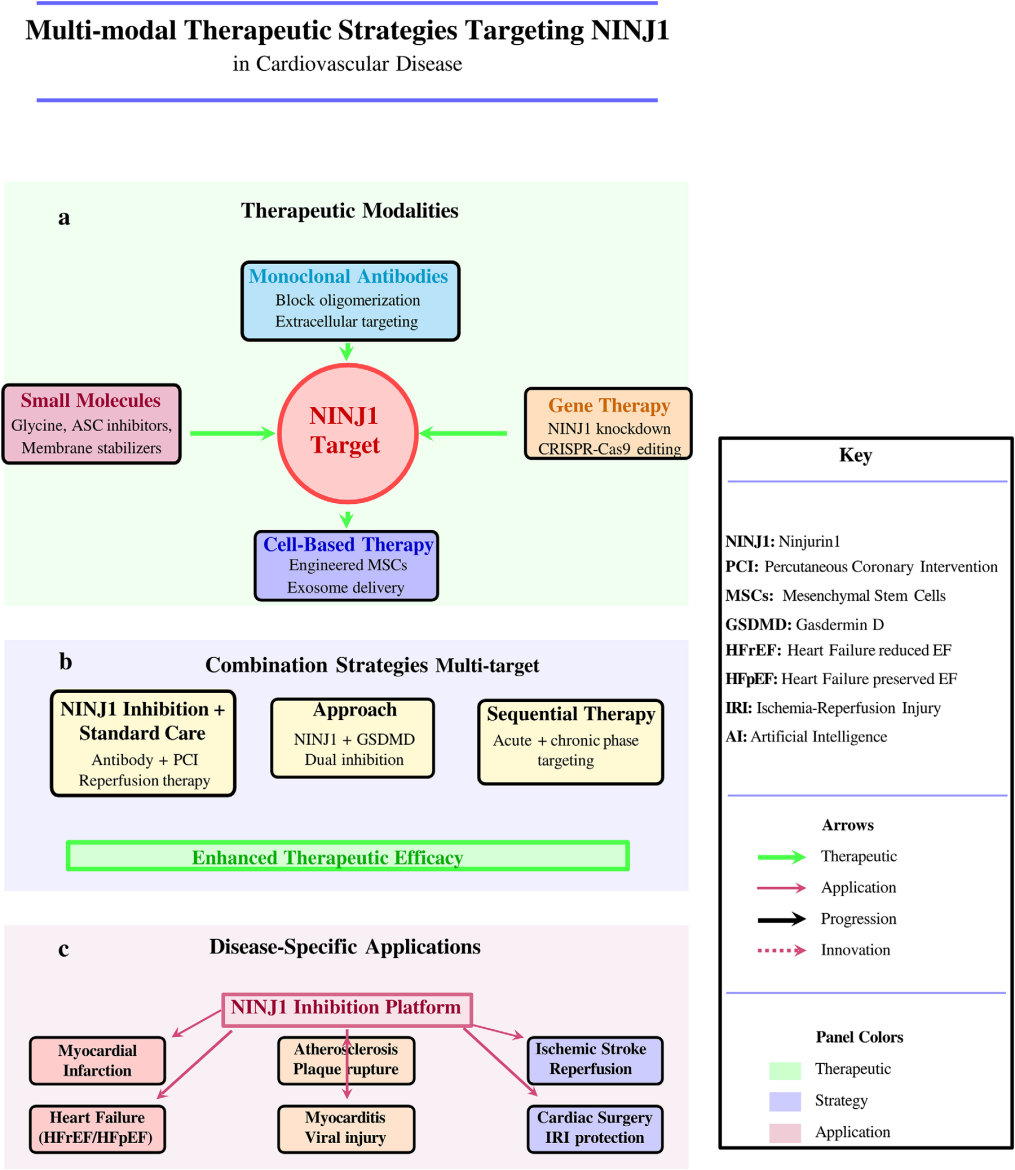
Multi‐modal therapeutic strategies targeting NINJ1 in cardiovascular disease. (A) *Therapeutic modalities*: Multiple approaches target NINJ1, including monoclonal antibodies that block extracellular oligomerization, small molecules such as glycine and ASC inhibitors that stabilize membranes, gene therapy approaches using NINJ1 knockdown or CRISPR‐Cas9 editing, and cell‐based therapies using engineered mesenchymal stem cells (MSCs) or exosome delivery systems. (B) *Combination strategies*: Enhanced therapeutic efficacy can be achieved through combining NINJ1 inhibition with standard care (antibody plus PCI), multi‐target approaches (dual NINJ1 and GSDMD inhibition), and sequential therapy targeting both acute and chronic phases of disease. (C) *Disease‐specific applications*: The NINJ1 inhibition platform shows promise across multiple cardiovascular conditions, including myocardial infarction, heart failure (both HFrEF and HFpEF), atherosclerosis with plaque rupture, viral myocarditis, ischemic stroke with reperfusion injury, and cardiac surgery‐ associated IRI protection.

Monoclonal antibodies targeting NINJ1 have demonstrated strong potential across multiple preclinical systems, offering a highly selective method for inhibiting NINJ1‐driven PMR without disrupting the protein's broader biological roles.^12^, ^15^ These antibodies are designed to recognise structural motifs essential for NINJ1 oligomerisation (Figure 6a)—particularly the interfaces required for filament assembly—thereby preventing the formation of higher‐order complexes that execute PMR.

Kayagaki et al. demonstrated that the anti‐NINJ1 monoclonal antibody D1 effectively inhibits PMR by binding to a C‐terminal epitope and sterically interfering with the lateral protein‒protein interactions required for NINJ1 oligomerisation.^23^ In murine hepatic IRI models, D1 administration prior to ischaemia‒reperfusion substantially reduced hepatocellular necrosis, serum ALT and aspartate aminotransferase elevations, and systemic DAMP release, providing robust proof of concept for therapeutic targeting of NINJ1. Key advantages of antibody‐based approaches include high target specificity, enabling selective oligomerisation blockade without affecting the autoinhibited dimer or monomeric NINJ1, and limited off‐target effects due to precise epitope engagement. However, significant challenges exist that must be acknowledged. Critically, the D1 antibody's pharmacokinetic profile is not amenable to chronic administration, as explicitly noted by Kayagaki et al. For CVDs requiring sustained NINJ1 inhibition—including atherosclerosis (years of treatment), chronic heart failure (indefinite treatment) or prevention of aneurysm progression (long‐term treatment)—this represents a substantial limitation. Potential strategies to address this include: (1) Fc engineering to extend serum half‐life through enhanced FcRn recycling; (2) development of antibody fragments (Fab, scFv, nanobodies) with improved tissue penetration balanced against shorter half‐lives; (3) conjugation to PEG (polyethylene glycol) or albumin‐binding domains; (4) local delivery approaches for accessible vascular targets; and (5) development of small molecule inhibitors suitable for oral chronic dosing.

### 5.2 Small molecule inhibitors

Recent studies demonstrating that glycine inhibits NINJ1 oligomerisation have delineated a previously unrecognised regulatory axis governing PMR and have broadened the therapeutic landscape for targeting lytic cell death.^105^

Glycine inhibits NINJ1 oligomerisation via an indirect mechanism that modulates membrane biophysical properties rather than through direct protein binding.^40^ As a zwitterionic osmolyte, glycine alters lipid packing density, membrane fluidity and protein‒lipid microdomain organisation in ways that disfavour the spatial alignment required for NINJ1 oligomer nucleation and propagation within the two‐dimensional membrane environment. This indirect, membrane‐based mechanism explains glycine's broad cytoprotective effects across multiple cell‐death pathways: by altering the membrane environment, glycine modulates the lipid context required for NINJ1 insertion and lateral oligomerisation. Critically, species‐specific differences in glycine sensitivity pose a significant translational challenge: murine NINJ1 is inhibited at low millimolar glycine concentrations (1‒5 mM), which, although supra‐physiological (normal plasma glycine ∼200‒400 µM), are potentially achievable through supplementation. However, human NINJ1 requires approximately 10‐fold higher glycine concentrations (10‒50 mM) for equivalent inhibition.^40^ Achieving such a concentration systemically would require massive glycine loading with uncertain safety and tolerability, substantially limiting translational potential.

Beyond glycine, den Hartigh et al. identified muscimol—a well‐characterised GABAA receptor agonist derived from Amanita muscaria mushrooms—as an inhibitor of NINJ1‐mediated PMR.^106^ Muscimol blocks NINJ1 oligomerisation through a mechanism that is clearly independent of GABAA receptor activity, as demonstrated by structure‒activity relationship studies showing that other GABAA agonists (including the benzodiazepine diazepam) do not recapitulate its protective effects. Muscimol's protective mechanism involves direct interaction with NINJ1 oligomerisation interfaces rather than with neuronal signalling. Crucially, the Fink group demonstrated that muscimol administration significantly reduced lethality in mice during LPS‐induced septic shock (survival improved from 20% to 65%, *p* < .01), providing important preclinical proof‐of‐concept for therapeutic NINJ1 inhibition in systemic inflammatory disease—evidence that glycine studies have not provided at comparable efficacy. This work positions muscimol and related compounds as promising therapeutic candidates for conditions involving excessive NINJ1‐mediated inflammation.

### 5.3 Peptide therapeutics: sNINJ1 mimetics

The recognition that sNINJ1 functions as an endogenous anti‐inflammatory and atheroprotective mediator has driven substantial interest in developing therapeutic peptides modeled on its N‐terminal NINJ1 anti‐inflammatory module (N‐NAM).^19^ Mimetic peptides such as ML56 and PN12 reproduce the protective activities of sNINJ1 in vivo, demonstrating robust suppression of macrophage‐driven inflammatory responses and significant attenuation of atherosclerotic plaque progression in multiple preclinical models.^19^, ^20^ These peptides exhibit pleiotropic mechanisms of action that converge to limit vascular inflammation and promote tissue homeostasis. The sNINJ1‐mimetic peptides containing the N‐NAM sequence (residues 26–37) exert their anti‐inflammatory effects via extracellular mechanisms, engaging cell‐surface targets to suppress inflammatory signalling. Given this extracellular site of action, cell‐penetrating peptide conjugation is unnecessary and would not confer a mechanistic advantage, as the therapeutic target is extracellular. The relevant delivery challenge is tissue penetration to sites of cardiovascular inflammation (atherosclerotic plaques, ischaemic myocardium, aneurysmal wall), not cellular penetration. Appropriate strategies include: nanoparticle formulation to enhance tissue penetration via the enhanced permeability and retention (EPR) effect in inflamed vasculature; targeting ligand conjugation (e.g., VCAM‐1‐binding peptides and collagen‐binding domains) for cardiovascular tissue specificity; and chemical modifications (D‐amino acid substitution, backbone cyclisation and PEGylation) to confer protease resistance and prolonged circulation half‐life.

In parallel, nanoparticle‐based delivery platforms enable targeted, controlled release of sNINJ1 mimetics.^107^ Lipid nanoparticles (LNPs), polymeric micelles and biodegradable PLGA nanoparticles can encapsulate N‐NAM peptides, protecting them from proteolytic degradation while enabling preferential accumulation in inflamed vascular regions through EPR effects or through ligand‐directed targeting.^108^, ^109^ Such formulations allow for precise tissue‐level delivery to atherosclerotic plaques, ischaemic myocardium or vascular lesions, thereby maximising therapeutic efficacy while minimising systemic exposure.

### 5.4 Gene therapy and RNA‐based approaches

Given NINJ1's complex and context‐dependent roles, cell‐type‐specific modulation may prove optimal. Gene therapy and RNA‐based approaches offer unprecedented precision for targeted intervention.

#### 5.4.1 MicroRNA therapeutics

The identification of microRNA‐125a‐5p as a negative regulator of NINJ1 suggests therapeutic potential.^60^ MicroRNA mimics could be delivered to suppress NINJ1 expression in specific cell types or tissues. Conversely, in contexts where NINJ1 downregulation is detrimental, anti‐miR oligonucleotides (antagomirs) targeting miR‐125a‐5p could preserve NINJ1 expression.^60^, ^110^ Advancements in oligonucleotide chemistry, including locked nucleic acids and cholesterol conjugation, have improved the stability and tissue uptake of microRNA therapeutics.^110^ For cardiovascular applications, conjugation to LNPs or to antibodies targeting endothelial‐ or macrophage‐specific markers could enable cell‐type‐specific delivery.^111^

#### 5.4.2 CRISPR‐based approaches

CRISPR‐based genome engineering provides a transformative platform for precise, heritable and potentially curative modulation of NINJ1 expression, enabling both mechanistic interrogation and therapeutic exploitation of NINJ1‐dependent PMR pathways.^85^ One major application involves ex vivo CRISPR–Cas9‐mediated disruption of NINJ1 (Figure 6a) in haematopoietic stem and progenitor cells (HSPCs), followed by autologous transplantation.^93^ Because HSPCs give rise to long‐lived myeloid populations—including monocytes, macrophages and dendritic cells—editing at the stem‐cell level could generate a durable immune compartment characterised by attenuated PMR and reduced DAMP efflux in chronic inflammatory diseases.^93^, ^112^ However, the complete ablation of NINJ1 must be approached cautiously. Emerging evidence suggests that total NINJ1 loss may impair appropriate termination of lytic forms of cell death, leading to intracellular retention of inflammatory cargo, defective efferocytosis, dysregulated macrophage programming and paradoxical increases in basal inflammation. Therefore, refined approaches such as allele‐specific disruption, partial knockdown or promoter engineering (rather than full knockout) may be necessary to preserve essential homeostatic functions while suppressing pathological PMR.

A second promising strategy involves targeted genomic insertion of constructs encoding sNINJ1 or engineered, stability‐enhanced sNINJ1 mimetics.^9^ Using homology‐directed repair or CRISPR‐associated transposases, cells could be reprogrammed to constitutively secrete anti‐inflammatory sNINJ1 fragments.^9^ This approach may overcome the pharmacokinetic limitations of peptide therapeutics by providing sustained, endogenous production of sNINJ1, enabling continuous modulation of inflammatory signalling pathways and interference with NINJ1‐dependent cell–cell adhesion events.

Furthermore, base editing and prime editing enable precise correction of single‐nucleotide variants in NINJ1 or NINJ2 that may confer increased susceptibility to atherosclerosis, vascular inflammation or enhanced inflammasome activation.^19^, ^89^ These editing modalities avoid double‐strand breaks and therefore reduce insertion–deletion formation, making them more suitable for correcting pathogenic alleles in non‐dividing tissues. Collectively, while genome editing holds tremendous therapeutic promise, numerous barriers—including off‐target genotoxicity, chromatin accessibility constraints, efficient delivery to cardiovascular tissues, long‐term immunogenicity of Cas nucleases, and stringent ethical limitations surrounding germline editing—must be overcome before clinical deployment becomes feasible.

### 5.5 Combination therapies

Emerging preclinical data support combining NINJ1‐targeted approaches with existing anti‐inflammatory treatments. Cui et al. showed in hepatic IRI models that combining anti‐NINJ1 antibodies with the NLRP3 inhibitor MCC950 provided better protection than either agent alone.^61^ This synergy is mechanistically plausible: MCC950 blocks upstream inflammasome activation, whereas anti‐NINJ1 prevents downstream PMR, thereby more effectively suppressing inflammation. Further studies in cardiac IRI models are needed. In addition to lowering lipids, statins decrease NINJ1 expression in atherosclerotic plaques through anti‐inflammatory effects. Jeon et al. found that statins reduced plaque NINJ1 in ApoE^‒/‒^ mice.^9^ Combining statins with NINJ1 inhibitors could provide additional atheroprotection, especially in patients with ongoing inflammation despite good lipid control. den Hartigh et al. demonstrated that muscimol, which inhibits NINJ1 oligomerisation, benefits inflammatory disease models.^106^ Combining muscimol with standard treatments such as corticosteroids or NSAIDs may allow dose reduction of each, maintaining efficacy while reducing side effects. Since sNINJ1 peptides (ML56 and PN12) reduce macrophage inflammation via mechanisms complementary to IL‐1β blockade, combining them with agents such as canakinumab (proven in the CANTOS trial for cardiovascular protection) is a logical approach.^2^ Although this combination has not yet been validated in preclinical studies, it is strongly justified. Nanoparticle delivery systems targeting macrophage NINJ1 could be paired with endothelial‐protective agents (such as NO donors or antioxidants) to optimise cell‐specific therapy while reducing off‐target effects. These combinations need thorough preclinical testing before clinical use, but the mechanistic basis and initial evidence support their potential.

### 5.6 Biomarker‐guided therapy

The successful clinical translation of NINJ1‐targeted therapeutics will require robust biomarker strategies to identify patients most likely to benefit from intervention. The landmark CANTOS trial established that targeting inflammation with canakinumab significantly reduces cardiovascular events, with patients achieving the greatest hsCRP reduction deriving the most clinical benefit.^2^ Similarly, the COLCOT trial demonstrated that low‐dose colchicine reduced cardiovascular events in patients after myocardial infarction, further supporting anti‐inflammatory approaches.^4^ These trials provide a framework for biomarker‐guided patient selection in NINJ1‐targeted studies.

Several candidate biomarkers merit consideration for patient stratification. Circulating sNINJ1, generated by MMP‐9‐mediated proteolytic cleavage, functions as an endogenous anti‐inflammatory mediator that competes with membrane NINJ1 for adhesion interactions, attenuating monocyte recruitment to atherosclerotic lesions.^9^ Low baseline sNINJ1 levels may identify patients who would benefit most from sNINJ1 mimetic therapy. DAMPs released during NINJ1‐dependent PMR represent direct readouts of lytic cell‐death activity.^13^ Key DAMP candidates include HMGB1, a nuclear alarmin implicated in myocardial IRI that activates TLR4/RAGE‐dependent inflammatory signalling,^113^ and S100A8/A9 (calprotectin), which predicts cardiovascular events independent of traditional risk factors.^114^ Patients with elevated composite DAMP scores likely have active NINJ1‐dependent inflammatory cell death, representing optimal candidates for anti‐NINJ1 intervention.

Inflammasome activation markers provide additional stratification opportunities. Circulating IL‐18, more specific than IL‐1β for inflammasome activation, is a strong predictor of cardiovascular death in patients with stable and unstable angina.^115^ Elevated IL‐18 indicates active NLRP3 inflammasome assembly driving GSDMD‐mediated pore formation and subsequent NINJ1‐dependent membrane rupture.^41^ Furthermore, 18F‐FDG PET quantifies arterial inflammation by detecting metabolically active macrophages and has been validated as a marker of plaque vulnerability responsive to anti‐inflammatory therapies.^116^

For clinical translation, we propose a phased approach: phase I safety evaluation establishing pharmacokinetics; phase II trials in STEMI patients (acute setting) and stable CAD patients with residual inflammatory risk (hsCRP > 2 mg/L) with biomarker stratification; and phase III cardiovascular outcomes trials powered for MACE with pre‐specified subgroup analyses based on sNINJ1 tertiles, DAMP composite scores and IL‐18 levels.^2^ This biomarker‐guided precision medicine approach maximises the probability of demonstrating clinical efficacy by enriching trial populations for patients most likely to benefit from NINJ1‐targeted intervention.

## 6 KNOWLEDGE GAPS AND FUTURE DIRECTIONS

Despite substantial advances in elucidating NINJ1's structural, biochemical and immunological roles, critical uncertainties remain that must be resolved to fully leverage NINJ1 as a therapeutic target in CVD. These gaps span cellular specificity, temporal regulation, metabolic integration, long‐term physiological consequences and translational feasibility.

A major limitation in current NINJ1 research is the predominance of in vitro work in isolated cell systems, which fails to capture the complex multicellular ecosystems that characterise cardiovascular pathology. The cardiovascular system comprises highly interactive networks of endothelial cells, smooth muscle cells, cardiomyocytes, fibroblasts, immune cells and extracellular matrix components. How NINJ1 functions within this interconnected landscape remains poorly defined. For example, NINJ1 expression on endothelial cells may influence the behaviour of adjacent smooth muscle cells or infiltrating macrophages by modulating adhesive interactions, paracrine cytokine gradients or extracellular vesicle communication. Similarly, cardiac fibroblasts and cardiomyocytes may exhibit unique NINJ1‐dependent signalling responses that shape post‐injury remodelling outcomes. Moreover, the extracellular matrix—rich in structural proteins, proteases, glycosaminoglycans and mechano‐transduction cues—likely influences NINJ1 oligomerisation and membrane insertion in ways not captured in reductionist models. Deciphering these cell‐type‐specific functions will require conditional knockout systems, CRISPR‐based lineage‐restricted perturbations, and next‐generation spatial transcriptomics and proteomics platforms capable of resolving NINJ1 activity in situ.^117^

NINJ1 function is likely temporally dynamic throughout the natural history of CVD. During the initiation phase of atherosclerosis, NINJ1 may contribute primarily to endothelial activation, monocyte recruitment and early immune–vascular crosstalk. In advanced plaques, its role may shift towards regulating pyroptotic or necroptotic membrane rupture and the release of DAMPs that amplify inflammation and promote necrotic core expansion. Following myocardial infarction, NINJ1 likely participates in acute injury responses characterised by widespread PMR, but may also influence chronic remodelling processes, including fibrosis, angiogenesis and immune cell resolution, over weeks to months. Longitudinal studies employing serial biopsies, advanced imaging modalities and single‐cell multi‐omic profiling are needed to define these phase‐specific functions and to identify time windows during which therapeutic modulation of NINJ1 would be optimally effective.

Accumulating evidence demonstrates profound sex‐specific differences in CVD incidence, immune activation patterns and treatment responses.^118^ Whether NINJ1 biology itself is sexually dimorphic remains unexamined. Sex hormones may regulate NINJ1 transcription, membrane localisation or cleavage by MMP‐9, thereby influencing sNINJ1 generation and downstream immunomodulatory effects. In addition, male and female immune systems exhibit distinct inflammatory thresholds, suggesting that NINJ1‐dependent PMR contributes differently to disease pathogenesis across sexes. Evaluating the sex‐specific efficacy, pharmacokinetics and toxicity of NINJ1‐targeted therapies will be essential for equitable clinical translation, necessitating balanced representation in preclinical and clinical study designs.

CVD, especially when associated with metabolic syndrome and diabetes, is increasingly recognised as a manifestation of ‘metaflammation’, in which metabolic dysregulation drives chronic innate immune activation.^119^ The interface between NINJ1 and metabolic pathways remains largely unexplored. Metabolic intermediates—including saturated fatty acids, glucose, ketone bodies or TCA (tricarboxylic acid) cycle metabolites—may regulate NINJ1 expression, oligomerisation thresholds or membrane‐binding capacity. Conversely, NINJ1‐mediated PMR may influence metabolic signalling by releasing metabolite‐rich DAMPs such as mitochondrial DNA, cardiolipin or ATP, thereby linking cell death to systemic metabolic inflammation. Determining whether established metabolic therapies, including SGLT2 inhibitors, metformin, GLP‐1 agonists or ketogenic diets, indirectly modulate NINJ1 activity could reveal new mechanistic insights and combinatorial therapeutic opportunities.

While short‐term pharmacological inhibition of NINJ1 has shown encouraging results in models of acute tissue injury, the long‐term consequences of sustained NINJ1 suppression remain unknown. Chronic inhibition could theoretically impair physiological lytic cell turnover, disrupt tissue homeostasis, or promote the accumulation of dysfunctional cells that would normally undergo PMR, thereby increasing oncogenic risk. Compensatory mechanisms may emerge, including upregulation of alternative membrane‐disrupting proteins or shifts in dominant cell‐death modalities. Comprehensive chronic‐exposure studies across multiple species, coupled with in‐depth immune phenotyping and long‐term oncological surveillance, will be critical for establishing safety profiles for NINJ1‐targeted therapies.

Although recent cryo‐EM structures have provided major advances in our understanding of NINJ1 architecture, key mechanistic questions remain unresolved. The molecular trigger that induces the transition from an autoinhibited dimer to an oligomerisation‐competent monomer during cell death remains unknown. Endogenous regulators—such as membrane lipids, ions, phosphorylation states, interacting proteins or mechanical stress—may operate as molecular switches that fine‐tune NINJ1 activation. Potential interactions with other membrane‐permeabilising effectors, such as gasdermins or MLKL, require detailed characterisation to determine whether these pathways converge, compete or synergise in various cell‐death contexts. Defining the ‘point of no return’ in NINJ1‐mediated PMR could guide therapeutic strategies aimed at halting cell‐death downstream of inflammasome or necroptosis activation. Addressing these questions will require integrative approaches combining live‐cell super‐resolution imaging, reconstituted lipid bilayer systems, biophysical force measurements, electrophysiology and high‐fidelity molecular dynamics simulations.^120^

Finally, successful translation of NINJ1‐targeted therapies from bench to bedside will depend on resolving several strategic and regulatory challenges.^85^ Biomarker‐driven patient selection will be essential to identify individuals most likely to respond to specific NINJ1‐directed interventions. Careful prioritisation will be needed to determine which cardiovascular indications—such as acute myocardial infarction, stroke, atherosclerosis or heart failure—offer the highest therapeutic leverage. Optimisation of dosing strategies, routes of administration (e.g., intravenous, subcutaneous, nanoparticle‐based delivery) and combination regimens with existing guideline‐directed therapies must be empirically defined. Early regulatory engagement in the discovery process, the establishment of multidisciplinary consortia, and the adoption of adaptive clinical trial frameworks will accelerate clinical translation while maintaining safety and scientific rigor. Collectively, addressing these knowledge gaps will be essential to realise the full therapeutic potential of targeting NINJ1 in cardiovascular medicine.

## 7 CONCLUSIONS

**FIGURE 7:**
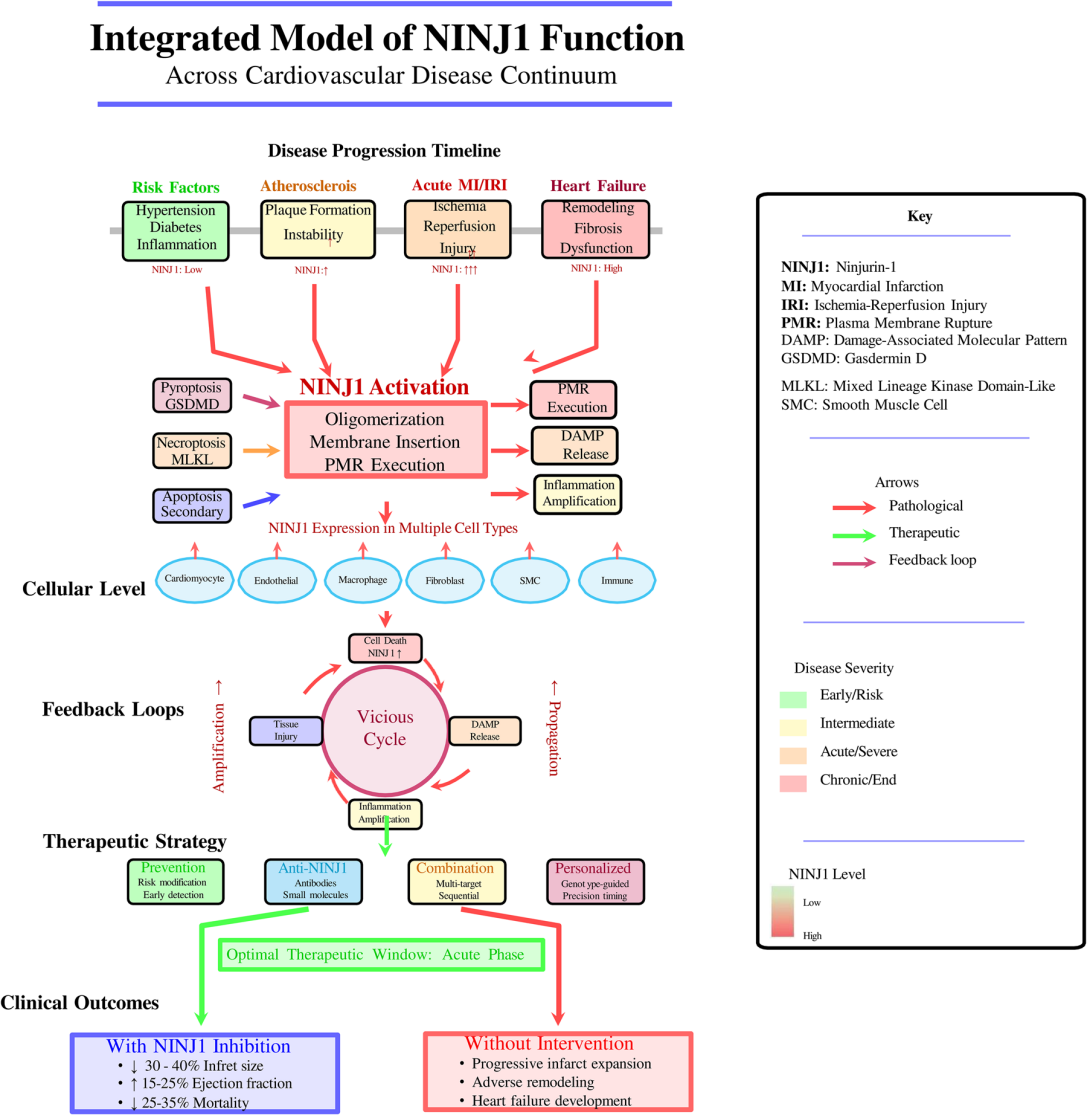
Integrated model of NINJ1 function across the cardiovascular disease continuum. This comprehensive single‐panel model illustrates the multifaceted role of NINJ1 throughout the progression of cardiovascular disease. Top—Disease progression timeline: The continuum begins with risk factors (hypertension, diabetes, inflam‐mation) where NINJ1 expression is low, progresses through atherosclerosis with plaque formation and instability (moderate NINJ1 upregulation), reaches peak NINJ1 expression during acute myocardial infarction and ischemia‐ reperfusion injury, and continues with sustained high NINJ1 levels during chronic heart failure with adverse remodeling and fibrosis. Central—NINJ1 mechanism hub: Multiple cell death pathways (pyroptosis via GSDMD, necroptosis via MLKL, and secondary apoptosis) converge on NINJ1 activation, which functions through oligomerization and membrane insertion, leading to plasma membrane rupture (PMR), damage‐associated molecular pattern (DAMP) release, and inflammatory amplification. Cellular level: NINJ1 is expressed across multiple cardiac cell types, including cardiomyocytes, endothelial cells, macrophages, fibroblasts, smooth muscle cells (SMC), and immune cells, coordinating tissue‐wide injury responses. Feedback loops: A self‐perpetuating vicious cycle emerges where NINJ1‐mediated cell death increases NINJ1 expression, leading to DAMP release, which amplifies inflammation, causing additional tissue injury that triggers more cell death, creating a pathological amplification and propagation system. Therapeutic strategy: Interventions span the entire continuum, including prevention through risk modification and early detection, targeted anti‐NINJ1 therapies using antibodies and small molecules, combination multi‐target and sequential approaches, and personalized, genotype‐guided precision timing. The optimal therapeutic window is identified during the acute phase. Clinical outcomes: With NINJ1 inhibition, patients can expect a 30‐40% reduction in infarct size, a 15‐25% improvement in ejection fraction, and a 25‐35% decrease in mortality. Without intervention, progressive infarct expansion, adverse remodeling, and heart failure development occur. This integrated model demonstrates how NINJ1 serves as a critical node connecting cellular mechanisms to tissue‐level pathology and provides a rational framework for therapeutic intervention across all stages of cardiovascular disease progression.

NINJ1 has evolved from an obscure adhesion molecule to a central effector in cardiovascular and vascular pathobiology, integrating molecular, cellular and tissue‐level mechanisms that govern inflammatory injury and repair. As synthesised in this review, NINJ1 occupies a uniquely conserved position as the executor of PMR downstream of pyroptosis, necroptosis and other lytic death pathways, thereby converting intracellular injury signals into extracellular inflammatory cues that propagate vascular dysfunction (Figure 7). The discovery that NINJ1 oligomerisation is tightly regulated by structural transitions and proteolytic cleavage underscores a sophisticated biological system in which membrane‐bound NINJ1 drives pro‐inflammatory PMR, whereas its MMP‐9‐derived soluble fragment exerts anti‐inflammatory and atheroprotective effects. This duality exemplifies the broader principle that cardiovascular homeostasis relies on finely tuned molecular switches that balance immune activation with resolution. Across vascular cell types, NINJ1 shapes disease processes through cell‐autonomous and non‐cell‐autonomous mechanisms, influencing endothelial barrier integrity, macrophage inflammatory programming, pericyte survival and cardiomyocyte death kinetics. These insights establish NINJ1 as a key convergence node linking DAMP release, leukocyte recruitment, microvascular destabilisation and adverse cardiac remodelling. They also highlight the need for context‐dependent therapeutic strategies, as the functional consequences of NINJ1 activation vary with disease stage, haemodynamic environment, metabolic state and cellular composition of the affected tissue. The mechanistic framework developed over the past several years now positions NINJ1 as a tractable therapeutic target. Pharmacologic inhibition of NINJ1 oligomerisation, augmentation of sNINJ1 activity and modulation of upstream regulatory pathways—such as the miR‐125a‐5p/NINJ1 axis—represent promising avenues across acute ischaemic injury, chronic vascular inflammation and metabolic vascular disease. Realising this potential will require concerted progress in structural elucidation, refinement of in vivo models with temporal and cell‐type resolution, identification of biomarkers for patient stratification, and development of targeted delivery systems capable of achieving high local specificity within the vasculature.

Collectively, NINJ1 research demonstrates how mechanistic dissection of a single membrane protein can reshape conceptual frameworks in cardiovascular immunology. By linking fundamental insights into membrane rupture with translational strategies for vascular protection, NINJ1 is poised to inform the next generation of therapies addressing the global burden of CVD.

## AUTHOR CONTRIBUTIONS

Muhammad Mamunur Rashid Mahib conceptualised the review, conducted the literature search and analysis, wrote the manuscript, reviewed and edited it, prepared the figures and approved the final version for submission.

## FUNDING INFORMATION

The authors received no specific funding for this work.

## ETHICS STATEMENT

The study did not require ethical approval.

## CONFLICT OF INTEREST STATEMENT

The author declares no conflicts of interest, financial or otherwise, related to the content of this review. This work was conducted independently without commercial sponsorship or competing financial interests that could be perceived as influencing the interpretation or presentation of the scientific literature discussed herein.

## Data Availability

This review article synthesises publicly available data from published research. No new experimental data were generated. All cited references are accessible through standard academic databases including PubMed, Web of Science and Google Scholar.
